# Expiration Date of Ready-to-Eat Salads: Effects on Microbial Load and Biochemical Attributes

**DOI:** 10.3390/foods10050941

**Published:** 2021-04-25

**Authors:** Panayiota Xylia, George Botsaris, Panagiotis Skandamis, Nikolaos Tzortzakis

**Affiliations:** 1 Department of Agricultural Sciences, Biotechnology and Food Science, Cyprus University of Technology, 3036 Limassol, Cyprus; pa.xylia@edu.cut.ac.cy (P.X.); george.botsaris@cut.ac.cy (G.B.); 2 Faculty of Food Science & Technology, Agricultural University of Athens, 54124 Athens, Greece; pskan@aua.gr

**Keywords:** food safety, foodborne pathogens, processes salads, respiration, polyphenols

## Abstract

When minimally processed vegetables reach their expiration date, expose an increased microbial load. This includes mainly spoilage microorganisms but also foodborne pathogens, thus affecting the quality and safety of highly consumed ready-to-eat salads. A total of 144 ready-to-eat salads from the Cypriot market were analyzed in an attempt to determine the effects of the expiration date on the microbial load and plant metabolic variables of the salads. Possible correlations between them were also investigated for the first time. Furthermore, the impacts of the season (winter, summer), salad producing companies and type of salad and/or their interactions with the tested parameters were investigated. Results revealed that the microbial load (mainly spoilage microorganisms, such as *Pseudomonas* spp., yeasts and molds) increased towards the end of the shelf life. The microbial load was differentiated among the five salad producers and/or the salad types, highlighting the importance of a common and safe sanitation-processing chain in the preparation of ready-to-eat salads. Summer was the season in which *Escherichia coli* counts were found to be higher for plain lettuce, while *Staphylococcus* spp. was increased numbers for the lettuce+endive/radicchio, lettuce+rocket and lettuce+chives type of salads. Additionally, an increased *Staphylococcus* spp. was observed for plain rocket salads in winter. All samples examined were found negative for *Salmonella enterica* and *Listeria monocytogenes.* Moreover, carbon dioxide production and damage indexes (hydrogen peroxide and lipid peroxidation) increased on expiration date on both winter and summer seasons, indicating plant tissue stress at the end of shelf life. These findings indicate that the expiration date and relevant shelf life of processed vegetables are important parameters to be considered when postharvest management is applied to these products, ensuring safety and quality.

## 1. Introduction

The importance of a balanced diet for the promotion of human health has led to the establishment of dietary guidelines (i.e., food wheel, MyPyramid, MyPlate), which aimed to present healthy eating habits with increased serving sizes of fruits and vegetables [1,2]. National organizations encourage people to increase fruits and vegetable intake [3]. However, increased consumption of fresh produce has been linked with the increase of food poisoning outbreaks [4,5,6]. Fruits and vegetables have been implicated in various outbreaks regarding the consumption of contaminated fresh produce, especially leafy vegetables, i.e., lettuce, spinach, cabbage and parsley [7,8,9,10].

Intensive cultivation of vegetables over the years for higher fresh produce yields has led to the appearance of increased food poisoning outbreaks linked with fresh produce consumption [7]. The probability of fresh produce contamination with foodborne pathogens is present along the food chain (from farm to consumer), and preharvest hazards play an important role in the prevalence of foodborne pathogens on fresh produce. Numerous routes have been previously reported, including water (of many sources), use of manure (poorly treated or even raw), insects, livestock and/or wild animals [7,11,12]. Concerns and challenges regarding food safety arise once pathogens are established in the environment.

Postharvest practices also provide sources of human pathogens that can possibly contaminate fresh produce, increasing the likelihood of food poisoning risks. During postharvest management, fresh produce, such as leafy vegetables, undergo processes, i.e., washing, shredding, chopping, slicing, peeling, which aim to reduce the microbial load of minimally processed vegetables and prepare them as ready-to-eat food [13]. However, along with mishandling and injured (surface damage), they can serve as sources of fresh produce contamination with foodborne pathogens lurking in the processing environment [14].

The main microflora of fruits and vegetables consists of spoilage bacteria, yeasts and molds accompanied by human pathogenic bacteria due to possible contamination through production (from cultivation to consumption) [15]. The main foodborne pathogens associated with fresh produce include EHEC *Escherichia coli*, *Salmonella* spp., *Listeria monocytogenes*, *Bacillus cereus*, *Campylobacter* spp., *Yersinia enterocolitica*, *Staphylococcus aureus* and *Clostridium botulinum* [5,15,16,17]. An infection with these pathogens could result in mild clinical symptoms, such as fever, headache, diarrhea, vomiting, abdominal pain and muscle cramps and/or more complex diseases/syndromes, including hemorrhagic colitis, hemolytic uremic syndrome, dysentery, septicemia, meningitis and even miscarriage [8,18]. Non-typhoidal *Salmonella* and Shiga-toxigenic *E. coli* were implicated in recent gastroenteritis outbreaks regarding the consumption of vegetables, sprouts, fruits and nuts [6,19,20,21,22].

It is known that adverse storage conditions (i.e., increased temperature) during postharvest handling and distribution can negatively affect organoleptic characteristics of leafy vegetables, i.e., appearance and aroma [23]. Moreover, the nutritional value of minimally processed vegetables might be adversely affected during processing. For instance, oxidation of phenolic content, degradation of vitamin C (ascorbic acid), loss of dietary fibers might take place due to preparing practices (i.e., cutting, shredding, washing) [24,25]. It has been previously mentioned that when minimally processed vegetables reach their expiration date, the increased microbial load was observed, including mainly spoilage microorganisms as well as foodborne pathogens [26,27,28].

The aim of this study was to evaluate the effects of the expiration date (OR “estimated expiration date”) on the microbial load and plant-associated parameters (phenolic content, antioxidants, carbon dioxide (CO_2_) production, damage indexes) of ready-to-eat salads collected in two seasons (winter and summer).

## 2. Materials and Methods

### 2.1. Sampling

A total of 144 ready-to-eat salads samples were randomly obtained from retail markets from the whole county of Cyprus (four cities of Larnaca, Limassol, Nicosia, and Paphos) in two sampling periods (seasons) in a one-year period: winter (January–February) and summer (July–August). Based on the sampled salads, seven different types of salads were collected, namely lettuce, lettuce + cabbage, lettuce + endive/radicchio, lettuce + rocket, lettuce + chives, rocket and other (lettuce + 2 or more ingredients). The ready-to-salads packaging/production in Cyprus is oriented in five enterprises (salads packaging units), namely salad ‘’producer" and are coded as producers A–E. For each period, sampling was performed once a week, and the collected samples were transferred in cool boxes to the laboratory within 2 h and immediately stored at a laboratory refrigerator (7 °C) for further analysis. To study the fresh produce perishability and sensitivity during storage in both foodborne pathogens and spoilage microorganisms, but also on their preservation/nutritive value, double samples were collected in each season, and half of them were directly analyzed as mentioned below, while the other half were stored at 7 °C until the expiration date (as indicated on each package, usually of 6 days).

Analyses performed included the determination of CO_2_ production (due to respiration process), polyphenol content, antioxidant activity, damage index (H_2_O_2_ production and lipid peroxidation), along with the examination of the microbial quality of samples (including spoilage and foodborne pathogens as described in Section 2.2).

An appropriate amount of fresh plant tissue (a representative portion from different parts of the salad) from each sample was collected and stored at −20 °C for microbiological analysis and the extraction of polyphenols. Modified atmosphere packaging (MAP) with single-layer oriented polypropylene (OPP) or double-layer polyethylene (PE) material was used by most salad producers/packagers. Fresh produce was sanitized with chlorine-based products in the washing process (approximately 2–3 ppm of free chlorine in the washing water), but sanitation before processing was not a common practice.

### 2.2. Microbiological Analyses

For the determination of the microbial quality of samples, the following parameters were assessed: total viable count (TVC), Enterobacteriaceae, coliforms, *E. coli*, *Staphylococcus* spp., *B. cereus*, LAB, *Pseudomonas* spp. and yeast and molds. Briefly, 1 g of plant tissue (the sampling weight was based on preliminary tests of 1–5–10 g of fresh tissue that showed no differences on microbiological quality and previous reports of Xylia et al. [29,30]) was homogenized in a ratio 1:10 (*w*/*v*) with maximum recovery diluent (MRD) (Merck, Darmstadt, Germany) in stomacher for 1 min and appropriate volume from decimal dilutions were inoculated to appropriate culture media: to determine TVC in plate count agar (Merck, Darmstadt, Germany) at 30 °C for 48 h; Enterobacteriaceae in violet red bile dextrose agar (VRBDA) (Merck, Darmstadt, Germany) at 37 °C for 24 h; coliforms and *E. coli* in coliform agar (Biolab, Hungary) at 37 °C for 24 h; *Staphylococcus* spp. in Baird-Parker agar (Merck, Darmstadt, Germany) supplemented with egg yolk tellurite emulsion (Merck, Darmstadt, Germany) at 37 °C for 24 h; *B. cereus* in Cereus Selective agar acc. to MOSSEL (MYP agar) (Merck, Darmstadt, Germany) supplemented with egg yolk (Merck, Darmstadt, Germany) and selective supplement (Merck, Darmstadt, Germany) at 30 °C for 48 h; *Pseudomonas* spp. in cetrimide–nalidixic acid (CN) agar for *Pseudomonas* (Biokar diagnostics, Allonne, France) at 37 °C for 48 h; lactic acid bacteria (LAB) in De Man, Rogosa and Sharpe agar (MRS agar) (Liofilchem S.r.l., Teramo, Italy) at 30 °C for 48 h and yeast and melds on Rose Bengal CAF agar (Liofilchem S.r.l., Teramo, Italy) at 25 °C for 5 days.

For the examination of antibiotic resistance (ability to produce *β*-lactamase) of typical isolated *E. coli* (blue) colonies from Coliforms agar (Biolab, Budapest, Hungary), a first subculture was performed on tryptone bile glucuronic agar (TBX agar) (HiMedia, Mumbai, India) with incubation at 37 °C for 24 h. Afterward, blue colonies from TBX were streaked on chromatic extended-spectrum *β*-lactamase (ESBL) agar (Liofilchem S.r.l., Teramo, Italy) and incubated at 37 °C for 24 h. ESBL producing *E. coli* isolates were identified by pink or purple colonies on ESBL agar.

#### 2.2.1. Isolation and Identification of *Salmonella* spp. and *Listeria* spp.

The isolation of *Salmonella* spp. was performed with the standard cultivation method as proposed by ISO [31] with some modifications based on preliminary trials, the available salad weight (~125–150 g/package) and previous records [30]. Briefly, 5 g of sample (as described above) were homogenized in 1:10 ratio with buffered peptone water (BPW) (Merck, Darmstadt, Germany) and incubated at 37 °C for 24 h. Afterward, 0.1 mL was added into 10 mL of Rappaport–Vassiliadis broth (RVS) (Merck, Darmstadt, Germany), which was incubated at 41.5 °C for 24 h. Finally, a loopful of RVS was streaked on xylose lysine deoxycholate agar (XLD agar) (Scharlau, Sentmenat, Spain) and incubated at 37 °C for 24 h. Typical red colonies with a black center were isolated, subcultured and incubated on brain heart infusion agar (BHI agar) (Biolab, Budapest, Hungary) at 37 °C for 24 h.

The isolation of *Listeria* spp. was carried out with the standard cultivation method as recommended by ISO [32], with modifications based on preliminary trials, the available salad weight (~125–150 g/package) and previous records [30]. Briefly, 5 g of sample (as described above) were homogenized in a 1:10 ratio with half-Fraser broth (Merck, Darmstadt, Germany) and incubated at 30 °C for 24 h. then 0.1 mL was added to 10 mL of full-Fraser broth (Merck, Darmstadt, Germany), which was incubated at 37 °C for 48 h. Finally, a loopful of full-Fraser broth was streaked on Augusti Ottaviani Listeria Selective agar (ALOA agar) (Merck, Darmstadt, Germany), which was incubated at 37 °C for 24 h. Typical blue-green colonies surrounded by an opaque halo (typical Listeria-like colonies) were isolated, subcultured and incubated on BHI agar (Biolab, Budapest, Hungary) at 37 °C for 24 h.

#### 2.2.2. Molecular Confirmation of *Salmonella enterica* and *Listeria monocytogenes*

DNA extraction was performed with heat lysis of previously isolated colonies. Briefly, one colony was diluted with 100 μL of sterile distilled water and heated for 20 min at 100 °C. Samples were centrifuged at 13,000× *g,* and the supernatant was stored at −20 °C until further use. *Listeria* spp. isolates were identified using real-time PCR for the amplification of a 274 bp fragment of the *prf*A gene with forward primer *prf*A LIP1 (5′-GATACAGAAACATCGGTTGGC-3′) and reverse primer *prf*A LIP2 (5′-GTGTAATCTTGATGCCATCAGG-3′) [33]. In both protocols, the template for real-time PCR assays was genomic DNA from heat-lysed cells (2 μL) and KAPA SYBR FAST qPCR master mix (KAPA Biosystems, Wilmington, MA, USA).

### 2.3. Carbon Dioxide Production

The estimation of CO_2_ produced due to the respiration of plant tissue was carried out using a dual gas analyzer (International Control Analyser Ltd., Kent, UK), as described previously [9]. In brief, prior to opening, the air of each package was sucked out for 60 s and results were expressed as a percentage (%) of CO_2_ production (value included the CO_2_ produced by respiration and the initially flushed CO_2_ in bags).

### 2.4. Polyphenol Content and Antioxidant Activity of Ready-to-Eat Salads

From each bag, pooled plant tissue (1 g) was collected and homogenized with 50% (*v/v*) methanol for 60 s. The process was further assisted with an ultrasound water bath (35 kHz frequency and 325 W power output) for 30 min. Homogenates were then centrifuged at 4600× *g* at 4 °C for 15 min, and the supernatants were stored at −20 °C until use.

Polyphenol content was estimated using the Folin–Ciocâlteu method at 755 nm, according to Tzortzakis et al. [34]. A calibration curve with gallic acid (Scharlau, Sentmenat, Spain) was used, and results were expressed as equivalents of gallic acid per g of fresh weight (mg of GAE/g Fw).

Three different methods were performed for the evaluation of the antioxidant activity of samples: 2,2-diphenyl-1-picrylhydrazyl (DPPH) assay, ferric reducing antioxidant power (FRAP) assay and 2.2′-azinobis-(ethylbenzothiazoline-6-sulfonic acid) (ABTS) assay. Methanolic extracts scavenging activity of the DPPH (Sigma-Aldrich, Steinheim, Germany) radical was examined with the procedure described by Chrysargyris et al. [35] at 517 nm, and results were expressed as mg of Trolox ((±)-6-hydroxy-2,5,7,8-tetramethylchroman-2carboxylic acid) per g of fresh weight (mg of Trolox/g Fw). The reducing ability of samples against Fe^3+^ at 593 nm was determined according to Chrysargyris et al. [35], and results were expressed as mg of Trolox per g of fresh weight (mg of Trolox/g Fw). Scavenging activity of sample’s methanolic extracts against ABTS^+^ (Sigma-Aldrich, Steinheim, Germany) radical at 734 nm was assessed as previously mentioned by Wojdylo et al. [36], and results were expressed as mg of Trolox per g of fresh tissue (mg of Trolox/g Fw).

### 2.5. Damage Index

Damage index was evaluated by the hydrogen peroxide (H_2_O_2_) production and lipid peroxidation on the examined plant tissue. The estimation of H_2_O_2_ production was carried out at 390 nm, as described by Loreto and Velikova [37]. A calibration curve with H_2_O_2_ was used, and results were expressed as μmol of H_2_O_2_ per g of fresh weight (μmol H_2_O_2_/g Fw). The 2-thiobarbituric acid reactive substances (TBARS) method was performed at 532 nm and 600 nm for the determination of samples lipid peroxidation according to de Azevedo Neto et al. [38], and results were expressed as nmol of malondialdehyde (MDA) per g of fresh weight (nmol MDA/g Fw).

### 2.6. Statistical Analysis

Statistical analysis was performed using IBM SPSS Statistics version 25, where the effect of season, salad producer and type of salad on the phenolic content, antioxidant activity, % CO_2_ production and damage index of samples was assessed. Data means were compared with one-way analysis of variance (ANOVA), and Tukey’s multiple range tests were calculated for the significant data *p* < 0.05. All measurements were done in duplicates. Differences between seasons were analyzed by the independent-samples *t*-test, while paired-samples *t*-test was performed for the determination of differences among purchase and expiration dates.

## 3. Results

### 3.1. Effects of Season

#### 3.1.1. Microbiological Analysis

##### Salad Producer

**Figure 1:**
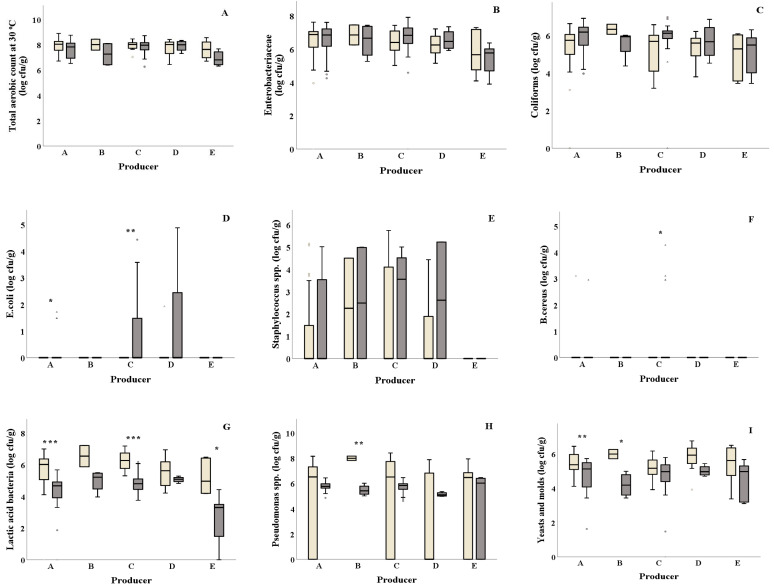
Effect of season on the microbiological quality (**A**–**I**) of ready-to-eat salads collected in winter (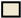) and summer (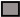) among salad producers/packagers (**A**–**E**). Results include all samples for each microorganism tested and are the mean value ± standard deviation. Each box contains 50 percent of cases, and whiskers represent the rest. The line across the inside of the box represents the median value. *, ** and *** indicate significant differences at *p* ≤ 5%, 1% and 0.1%.

Significant differences of *E. coli* were reported from salad producers A and C, where both producers showed higher values in summer (0.32 and 0.68 log cfu/g, respectively) compared to winter (0.00 log cfu/g, both) (Figure 1D and Table 1). Higher *B. cereus* values were observed in summer compared to winter for salad producer C (0.39 and 0.00 log cfu/g, respectively) (Figure 1 F), while salad producer B showed higher *Pseudomonas* spp. counts in winter compared to summer (8.00 and 5.47 log cfu/g, respectively) (Figure 1H). Salad producers A, C and E presented significantly lower LAB values in summer (4.13, 4.84 and 2.67 log cfu/g, respectively) compared to winter (5.66, 6.27 and 5.20 log cfu/g, respectively) (Figure 1G). Yeasts and molds were found to be significantly higher (*p* = 0.002) in winter for salad producer A compared to summer (Figure 1I). Similarly, producer B showed higher yeasts and molds counts in winter (5.99 and 4.20 log cfu/g, respectively). No significant differences for TVC, Enterobacteriaceae, coliforms and *Staphylococcus* spp. were observed among samples collected in winter and summer for all salad producers (Figure 1A–C,E and Table 1).

##### Type of Salad

**Figure 2:**
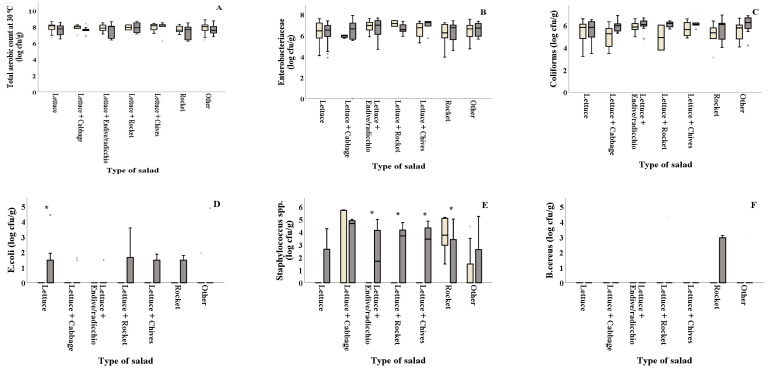

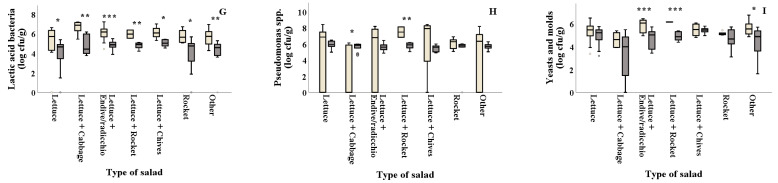
Microbiological quality (**A**–**I**) of different types of ready-to-eat salads collected in winter (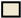) and summer (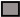). Results include only positive samples for each microorganism tested and are the mean value ± standard deviation. Other = lettuce + 2 or more ingredients. Each box contains 50 percent of cases, and whiskers represent the rest. The line across the inside of the box represents the median value. *, ** and *** indicate significant differences at *p* ≤ 5%, 1% and 0.1%.

As shown in Figure 2D and Table 2, *E. coli* counts were reported significantly (*p* = 0.044) higher in summer for salads containing plain lettuce. The combinations of lettuce with other types of leafy vegetables (lettuce + endive/radicchio, lettuce + rocket and lettuce+chives) showed significantly higher *Staphylococcus* spp. counts in summer (2.10, 2.55 and 2.67 log cfu/g, respectively) compared to winter (0.32, 0.00 and 0.00 log cfu/g, respectively), while rocket presented higher values (*p* = 0.032) in winter compared to summer (Figure 2E). LAB on all types of salads was found in decreased numbers in summer (ranging from 3.98 to 5.02 log cfu/g) compared to winter (ranging from 5.21 to 6.63 log cfu/g) (Figure 2G). Samples of lettuce + cabbage collected in summer showed significantly higher *Pseudomonas* spp. values compared to those collected in winter (5.61 and 2.00 log cfu/g, respectively), while the opposite was evidenced for the lettuce + rocket samples that showed higher values in winter compared to summer (7.47 and 5.78 log cfu/g) (Figure 2H). Moreover, yeasts and molds were found in significantly decreased numbers in samples of lettuce + endive/radicchio, lettuce+rocket and other (lettuce + two or more ingredients), collected in winter (4.74, 4.91 and 4.39 log cfu/g, respectively) compared to the ones collected in summer (5.84, 6.16 and 5.58 log cfu/g, respectively) (Figure 2I). TVC, Enterobacteriaceae, coliforms and *B. cereus* counts were not significantly different (*p* > 0.05) among samples collected in winter and summer for all types of salad, as shown in Figure 2A–C,F and Table 2.

#### 3.1.2. Total Phenols Content, Antioxidants, CO_2_, H_2_O_2_ and Lipid Peroxidation

##### Salad Producer

**Figure 3:**
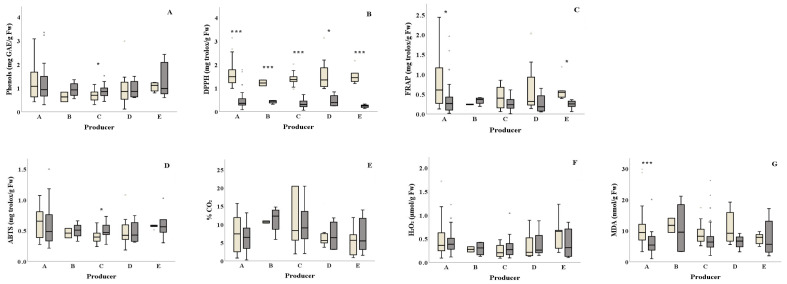
Effect of season on the total phenolic content, antioxidants, % CO_2_ and damage index (H_2_O_2_ and lipid peroxidation) (**A**–**G**) of ready-to-eat salads collected in winter (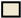) and summer (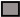) among salad producers/packagers (A, B, C, D, and E). Results include all samples for each microorganism tested and are the mean value ± standard deviation. Each box contains 50 percent of cases, and whiskers represent the rest. The line across the inside of the box represents the median value. * and *** indicate significant differences at *p* ≤ 5% and 0.1%.

Total phenols content was found higher for producer C in summer compared to winter (0.86 and 0.69 mg GAE/g Fw) (Figure 3A and Table 1). Samples from all salad producers collected in winter showed significantly higher DPPH antioxidant values (DPPH: ranging from 1.22 to 1.60 mg Trolox/g Fw, respectively) compared to the ones collected in summer (DPPH: ranging from 0.23 to 0.51 mg Trolox/g Fw, respectively) (Figure 3Β and Table 1). Producer E samples showed significantly lower FRAP activity (*p* = 0.015) in summer compared to winter (Figure 3C). Similarly, samples from producer A presented lower FRAP activity in summer compared to the ones collected in winter. Samples from producer C presented higher ABTS activity in summer compared to winter (ABTS: 0.49 and 0.41 mg Trolox/g Fw, respectively) (Figure 3D). Higher lipid peroxidation was observed in winter for producer A samples compared to summer (10.56 and 6.21 nmol MDA/g Fw, respectively) (Figure 3G). The H_2_O_2_ production and% CO_2_ did not differ among the examined producers for both seasons (Figure 3E,F and Table 1).

##### Type of Salad

**Figure 4:**
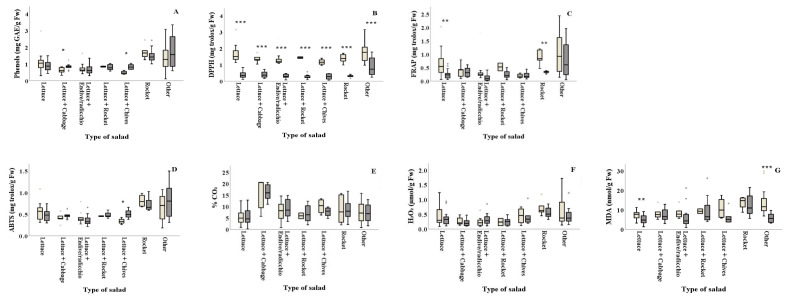
Effect of season on the total phenolic content, antioxidants, % CO_2_ and damage index (H_2_O_2_ and lipid peroxidation) (**A**–**G**) of ready-to-eat salads collected in winter (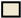) and summer (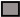) among types of salads. Results include all samples for each microorganism tested and are the mean value ± standard deviation. Other = lettuce + 2 or more ingredients. Each box contains 50 percent of cases, and whiskers represent the rest. The line across the inside of the box represents the median value. *, ** and *** indicate significant differences at *p* ≤ 5%, 1% and 0.1%.

Salad types of lettuce+cabbage and lettuce+chives revealed higher phenolic content in summer (0.85 and 0.84 mg GAE/g Fw, respectively) compared to winter (0.62 and 0.49 mg GAE/g Fw, respectively) (Figure 4A and Table 2). The DPPH assay revealed that antioxidant content of all types of salad significantly differed between the two seasons, with summer (ranging from 0.27 to 0.89 mg Trolox/g Fw, respectively) showing lower values than winter (ranging from 1.17 to 1.77 mg Trolox/g Fw, respectively) (Figure 4B and Table 2). Plain lettuce and plain rocket presented higher antioxidant activity in winter (FRAP: 0.64 and 0.88 mg Trolox/g Fw, respectively) compared to summer (FRAP: 0.25 and 0.33 mg Trolox/g Fw, respectively) (Figure 4C). On the other hand, lettuce+chives significantly decreased the ABTS antioxidant activity in winter compared to summer (ABTS: 0.34 and 0.51 mg Trolox/g Fw, respectively) (Figure 4D). Lipid peroxidation was found to be significantly higher in winter for plain lettuce (*p* = 0.002) and lettuce+two or more ingredients (other) (*p* = 0.003) (7.49 and 13.50 nmol MDA/g Fw, respectively) compared to summer (5.01 and 5.85 nmol MDA/g Fw, respectively) (Figure 4G). No significant differences (*p* > 0.05) were reported for% CO_2_ and H_2_O_2_ production among samples collected in winter and summer for all types of salad, as illustrated in Figure 4E,F and Table 2.

### 3.2. Effects of Shelf Life

#### 3.2.1. Microbiological Analysis

##### Salad Producer

*Purchase (actual) vs. Expiration date in winter and summer*: The effect of the storage period of purchase and expiration date on microbial load on different salad producers are presented in Figure S1 and Table 3. Salads from producer A exhibited significantly higher TVC numbers at the end of their shelf life in winter than the expiration date in summer (Figure S1). During summer, the polynomial curve with concave upward was described by y = 0.0556x^2^ − 0.2409x + 7.3881; R^2^ = 0.92, while the relevant curve in winter was y = 0.0065x^2^ + 0.077x + 7.5665; R^2^ = 0.49. Moreover, in winter, salads from producer A on their expiration date exhibited higher Enterobacteriaceae with polynomial curve (concave downward) described by y = −0.1334x^2^ + 1.1601x + 4.78; R^2^ = 0.92 (Figure S1), and increased LAB counts with polynomial curve and concave downward described by y = −0.0995x^2^ + 0.8922x + 4.387; R^2^ = 0.80 (Figure S1). Summer was the season in which increased *Staphylococcus* spp. with a polynomial curve (concave upward) described by y = 0.092x^2^ − 0.3665x + 0.7854; R^2^ = 0.48 for salads from producer A on an expiration date (Figure S1). Additionally, during the summer period, salads from producer B on an expiration date revealed decreased yeasts and mold numbers with polynomial curve and concave downward being described by y = −0.3454x^2^ + 2.8219x − 0.4864; R^2^ = 1.00 (Figure S1). Samples from all salad producers collected throughout shelf life did not present significant differences among seasons for coliforms, *E. coli*, *B. cereus*, and *Pseudomonas* spp. (Figure S1). Due to the pre-enrichment and enrichment of samples, the presence/absence of *L. monocytogenes* in all samples was examined. Presumptive colonies from three samples (3 salads) were isolated, but when PCR tested, none of them was identified as *L. monocytogenes.*

##### Type of Salad

*Purchase (actual) vs. Expiration date in winter and summer*: The effect of the storage period of purchase and expiration date on microbial load on different types of salads are presented in Figure S2 and Table 4. Expiration date TVC numbers were found higher in both seasons for lettuce+endive/radicchio (8.13 and 8.01 log cfu/g for winter and summer, respectively) compared to purchase day (7.61 and 7.27 log cfu/g for winter and summer, respectively) (Table 4) During winter, in case of lettuce+endive/radicchio the polynomial curve with concave upward is described by y = 0.0694x^2^ − 0.4229x + 8.156; R^2^ = 0.68, while the relevant curve (concave downward) in summer is described by y = −0.0287x^2^ + 0.5487x + 5.8757; R^2^ = 0.86 (Figure S2). During summer, increased TVC numbers were observed for lettuce+rocket on the product expiration date (*p* = 0.012; 8.45 log cfu/g), whereas rocket and lettuce+two or more ingredients (other) presented higher expiration TVC counts in winter (7.87 and 8.22 log cfu/g, respectively) (Table 4). The polynomial curve with concave upward for lettuce+rocket is described by y = 0.1238x + 7.5249; R^2^ = 1.00, while the relevant curves for rocket and other are described by y = 0.0384x^2^ − 0.1831x + 7.5846; R^2^ = 1.00 and y = 0.0091x^2^ + 0.1099x + 7.2934; R^2^ = 0.85, respectively (Figure S2). Expiration Enterobacteriaceae numbers were found increased on lettuce+endive/radicchio on both seasons (7.26 and 7.09 log cfu/g for winter and summer, respectively) compared to purchase day (6.51 and 6.39 log cfu/g for winter and summer, respectively) and the polynomial curve with concave upward for winter is described by y = 0.032x^2^ − 0.0589x + 6.4607; R^2^ = 0.99, while the relevant curve (concave downward) for summer is described by y = −0.1721x^2^ + 1.7325x + 2.9305; R^2^ = 0.95 (Figure S2). Winter was the season in which plain lettuce and lettuce+endive/radicchio exhibited significantly higher coliform counts on expiration date compared to purchase day (Table 4), and the relevant polynomial curves are described in Figure S12A,C. *Pseudomonas* spp. and yeasts and molds counts were found to be significantly higher on an expiration date for lettuce+rocket in summer (5.85 and 5.24 log cfu/g, respectively) compared to purchase day (5.71 and 5.24 log cfu/g, respectively), while on the same season increased LAB numbers were observed for lettuce+rocket on purchase day compared to the expiration date (4.90 and 4.68 log cfu/g, respectively) (Figure S2 and Table 4). During summer for lettuce+rocket, the polynomial curves for *Pseudomonas* spp. and yeasts and molds with concave upward are described by y = 0.0403x^2^ + 5.6089x; R^2^ = 1.00 and y = 0.1393x^2^ − 0.9671x + 6.028; R^2^ = 1.00, respectively, while the relevant curve for LAB is described by y = 0.1141x^2^ − 1.0079x + 6.6222; R^2^ = 1.00 (Figure S2). No significant differences were observed between the day of purchase and the expiration date of salads among seasons for *E. coli*, *Staphylococcus* spp. and *B. cereus* (Figure S2). *L. monocytogenes* in all samples were negative-tested, as described above.

#### 3.2.2. Total Phenolic Content, Antioxidants, CO_2_, H_2_O_2_ and Lipid Peroxidation

##### Salad Producer

*Purchase (actual) vs. Expiration date in winter and summer*: The effect of the storage period of purchase and expiration date on plant-related parameters on different salad producers is presented in Figure S3 and Table 3. Increased expiration CO_2_ production was reported for producer A on both seasons (10.52 and 8.95% CO_2_ for winter and summer, respectively). During winter, the polynomial curve with concave upward is described by y = 0.3216x^2^ − 1.1189x + 4.4455; R^2^ = 0.44, while the relevant curve (concave downward) in summer is described by y = −0.2193x^2^ + 2.5629x + 1.5943; R^2^ = 0.94 (Figure S3). For producer C, increased CO_2_ production was also observed on the expiration date for both seasons (14.25 and 12.09% CO_2_ for winter and summer, respectively), and the relevant polynomial curves (concave upward and downward) are described in Figure S3. Moreover, salads from producer E presented higher CO_2_ production on their expiration date on both seasons (7.73 and 10.31% CO_2_ for winter and summer, respectively) compared to purchase day (3.24 and 3.44% CO_2_ for winter and summer, respectively). The polynomial curve with concave upward for winter is described by y = 0.8554x^2^ − 4.9645x + 6.765; R^2^ = 0.96, while the relevant curve for summer is described by y = 1.56x^2^ − 11.472x + 23.244; R^2^ = 0.97 (Figure S3). Higher expiration H_2_O_2_ levels were reported for samples from producers A and C in both seasons (A: 10.04 and 5.86 μmol H_2_O_2_/g Fw; C: 6.97 and 7.20 μmol H_2_O_2_/g Fw for winter and summer, respectively), and the relevant polynomial curves are described in Figure S3. Furthermore, in winter, samples from producers D and E presented higher expiration H_2_O_2_ levels compared to purchase day. During winter, the polynomial curve with concave upward for producer D is described by y = 0.0519x^2^ − 0.3925x + 0.8563; R^2^ = 0.99, while the relevant curve for producer E is described by y = 0.0207x^2^ − 0.0554x + 0.4204; R^2^ = 0.52 (Figure S3). Increased MDA levels were reported on the expiration dates for samples from producers A and C in both seasons (Figure S3). Samples collected in winter from producers D and E presented higher MDA levels on their expiration date (12.47 and 7.20 nmol MDA/g Fw, respectively) compared to purchase day (0.37 and 0.85 nmol MDA/g Fw, respectively), and the relevant polynomial curves are described in Figure S3. No significant differences (*p* > 0.05) were observed between the day of purchase and the expiration date of salads among producers in both seasons for their phenolic content and antioxidant capacity (with DPPH, FRAP and ABTS assays) (Figure S3).

##### Type of Salad

*Purchase (actual) vs. Expiration date in winter and summer*: The effect of the storage period of purchase and expiration date on plant-related parameters on different types of salads are presented in Figure S4 and Table 4. Increased phenolic content was observed on the expiration date in summer (*p* = 0.010) for lettuce+chives compared to purchase day (0.97 and 0.70 mg GAE/g Fw, respectively) and the polynomial curve with concave upward is described by y = 0.0398x^2^ − 0.2518x + 1.0533; R^2^ = 1.00 (Figure S4 and Table 4). During winter, increased FRAP antioxidant activity was reported on the expiration date for lettuce+cabbage compared to purchase day, while plain rocket showed higher FRAP on the expiration date in summer compared to purchase day (Figure S4). The relevant polynomial curve with concave upward for lettuce+cabbage is described by y = 0.0047x^2^ + 0.0384x + 0.1125; R^2^ = 0.63, while the respective curve for rocket is described by y = 0.0112x^2^ − 0.0713x + 0.3847; R^2^ = 0.73 (Figure S4).

Increased CO_2_ production was observed for plain lettuce and lettuce+radicchio for both seasons on expiration date compared to purchase day, and the relevant polynomial curves are described in Figure S4. Summer was the season in which lettuce+rocket and lettuce+chives presented higher CO_2_ production on the expiration date (9.25 and 9.26% CO_2_, respectively) compared to purchase day (4.58 and 6.01% CO_2_, respectively). During summer, the polynomial curve for lettuce+rocket with concave downward is described by y = −0.5253x^2^ + 5.6869x − 5.9625; R^2^ = 1.00, whereas the relevant curve for lettuce+chives is described by y = −0.0406x^2^ + 0.6438x + 3.965; R^2^ = 1.00 (Figure S4). Higher expiration CO_2_ production was reported significantly higher on the expiration date for plain rocket and lettuce+two or more ingredients (other) (*p* = 0.004 and 0.001, respectively) (14.25 and 10.31% CO_2_, respectively) compared to purchase day (2.84 and 4.53% CO_2_, respectively) in winter (Figure S4 and Table 4). During winter, the polynomial curve for rocket with concave upward is described by y = 0.2208x^2^ + 0.6242x + 2.555; R^2^ = 1.00, whereas the relevant curve for lettuce+two or more ingredients (other) is described by y = 0.343x^2^ − 1.313x + 5.0301; R^2^ = 0.65 (Figure S4).

Plain lettuce’s, lettuce+cabbage and lettuce+endive/radicchio expiration H_2_O_2_ levels were found significantly higher on both seasons compared to purchase day, and their relevant polynomial curves are described in Figure S4 and Table 4). H_2_O_2_ levels for lettuce+chives were increased in summer on the expiration date than the purchase day (4.67 and 0.30 μmol H_2_O_2_/g Fw, respectively), and the polynomial curve with concave upward is described by y = 0.0532x^2^ − 0.3693x + 0.861; R^2^ = 1.00 (Figure S4). Plain rocket’s and lettuce+two or more ingredients (other) expiration H_2_O_2_ levels were found significantly increased on both seasons compared to purchase day, and their relevant polynomial curves are described in Figure S4 and Table 4.

Plain lettuce’s, lettuce+cabbage and lettuce+endive lipid peroxidation levels were found significantly increased on both seasons on the expiration date (lettuce: 8.08 and 4.91 nmol MDA/g Fw; lettuce+cabbage: 10.39 and 7.14 nmol MDA/g Fw; lettuce+endive/radicchio: 8.54 and 5.95 nmol MDA/g Fw for winter and summer, respectively) compared to purchase day (lettuce: 0.49 and 0.27 nmol MDA/g Fw; lettuce+cabbage: 0.24 and 0.19 nmol MDA/g Fw; lettuce+endive/radicchio: 0.21 and 0.36 nmol MDA/g Fw for winter and summer, respectively) and their relevant polynomial curves are described in Figure S4 and Table 4). Plain rocket’s expiration MDA levels were found significantly increased on both seasons (15.27 and 15.19 nmol MDA/g Fw for winter and summer, respectively), and the polynomial curve for winter with concave upward is described by y = 0.9881x^2^ − 7.2301x + 19.413; R^2^ = 1.00, whereas the relevant curve for summer is described by y = 0.1919x^2^ − 0.6568x + 4.8891; R^2^ = 1.00 (Figure S4 and Table 4). Similarly, lipid peroxidation levels for lettuce+two or more ingredients (other) were increased on expiration date for both seasons (14.54 and 5.80 nmol MDA/g Fw winter and summer, respectively) and the polynomial curve for winter with concave downward is described by y = −0.3059x^2^ + 2.4755x + 9.6229; R^2^ = 0.29, whereas the relevant curve for summer is described by y = −0.0791x^2^ + 0.4891x + 5.7124; R^2^ = 1.00 (Figure S4).

No significant differences (*p* > 0.05) were observed between the day of purchase and the expiration date of salads among seasons for their antioxidant activity (with DPPH and ABTS assays) (Figure S4).

## 4. Discussion

Higher *E. coli* populations were observed for samples from salad producers A and C in summer compared to winter samples, while samples from producer C showed increased *B. cereus* counts in summer. It has been previously mentioned that *Bacillus* spp. and *Pseudomonas* spp. (including *Bacillus mojavensis*, *Bacillus megaterium* and *P. fluorescens*) have been isolated from ready-to-eat salads [39]. The presence of these bacteria may accelerate the degradation of vegetables, or they can antagonize foodborne pathogens, such as *Listeria monocytogenes* and *Salmonella enterica* in that environment [39]. During winter, higher levels of spoilage microorganisms, such as yeasts and molds, were reported for producers A and B. Furthermore, *Pseudomonas* spp. counts were increased for producer B in winter, while LAB was found higher for producers A, C and E in the same season. The presence of LAB was evident since the beginning of the processing of ready-to-eat vegetables, and increased numbers were reported after seven days of storage at 4 °C for sliced cabbage (air packaging), iceberg lettuce chopped (MAP), mixed endive, radicchio and “lollo rosso” lettuce (MAP) [40]. This may suggest that LAB is part of the endogenous and epiphytic microflora of raw fresh produce. In a study conducted in Italy, no significant difference in yeasts and mold populations of ready-to-eat salads (rocket, baby leaf lettuce and lamb’s lettuce) was reported among spring and summer [41]. No significant differences were observed for TVC, Enterobacteriaceae, coliforms and *Staphylococcus* spp. among seasons for all producers. On the other hand, aerobic psychotropic microorganisms were found in high numbers (up to 8.5 log cfu/g) in ready-to-eat salads collected in summer in Portugal [39]. The differences in the microbial load between seasons might be attributed to the different climatic conditions in each geographic area of cultivation. For instance, in Italy and Cyprus, as in many other Mediterranean countries, autumn and winter are characterized by rainfall (high moisture levels) and relatively low temperatures [26,30]. These observations might partially explain the high levels of psychrotrophic microorganisms (i.e., *Pseudomonas* spp., LAB, yeasts and molds) reported in winter (compared to summer) in the present study.

Total phenolic content was increased in summer for samples from producer C, while antioxidants were increased for all salad producers in winter. Caponigro et al. [26] reported higher average visual quality in winter and spring compared to summer and autumn. These findings might suggest less phenolic oxidation levels and other degradative processes that can compromise the nutritional value (phenols, antioxidants) of these products. Lipid peroxidation increased for samples collected from producer A in summer compared to winter. Kang and Saltveit [42] have previously mentioned that wounding of plant tissue (i.e., from cutting) can induce increased antioxidant activity in romaine and iceberg lettuce. No differences were observed for CO_2_ production and H_2_O_2_ levels among samples for all producers among seasons.

Summer was the season in which *E. coli* counts were found to be higher for plain lettuce. *Staphylococcus* spp. was found in increased numbers in summer for the lettuce + endive/radicchio, lettuce + rocket and lettuce + chives type of salads, while for plain rocket increased *Staphylococcus* spp. was reported in winter. Bell et al. [23] reported significantly increased microbial load (total aerobic counts) of rocket salad during shelf life. Decreased LAB populations were observed in summer for all types of salad, while *Pseudomonas* spp. was found in higher numbers for lettuce + cabbage in summer, while higher counts were also reported in winter for lettuce+rocket. High yeasts and mold counts were observed in winter for lettuce + endive/radicchio, lettuce + rocket and lettuce + two or more ingredients (other). De Corato [41] reported that lettuce salad presented lower yeasts and mold counts compared to rocket and lamb’s lettuce. No significant differences were observed for TVC, Enterobacteriaceae, coliforms and *B. cereus* for all producers among seasons. Santos et al. [39] reported increased aerobic psychrotrophic microorganisms for ready-to-eat salads (romaine lettuce and mixed vegetable salads) collected in summer compared to spinach samples in the same season. As previously mentioned, the combination of lettuce with other leafy vegetables presented increased *E. coli* counts as well as antioxidants (DPPH, FRAP), while at the same time, TVC, Enterobacteriaceae and coliforms were found in lower levels [30]. These variations might be attributed to the different microbial load of each vegetable used in the salad mix as well as the processing applied each time.

Increased phenolic content of the lettuce+cabbage and lettuce+chives was observed in summer, while the high antioxidant capacity of samples was observed in winter. Moreover, plain lettuce and rocket showed higher antioxidant activity in winter (as shown by the FRAP assay). Higher lipid peroxidation was reported in winter for plain lettuce and lettuce+two or more ingredients (other). Ferrante et al. [43] reported higher lipid peroxidation values on fresh-cut lamb’s lettuce leaves compared to intact ones when stored at 4 °C up to eight days (up to 51 nmol MDA/g Fw), suggesting that processing, such as cutting along with storage duration and conditions induce plant stress. No differences were observed for CO_2_ production and H_2_O_2_ levels among samples for all producers among seasons. On the other hand, in another study, high CO_2_ production was reported for rocket salads stored at 5 and 10 °C, and this could be attributed to the high respiration rate of rocket as well as to the abusive storage temperatures (optimum storage conditions for rocket: 0 °C with 95–100% RH) [44].

The expiration date of ready-to-eat salads is a matter of high importance since minimally processed vegetables reaching the maximum of their shelf life start to present defects, such as wilting, browning (loss of green color), development of off-odors and off-flavors that reduce the product’s acceptance from consumers [28]. Furthermore, increased spoilage (mostly) and pathogenic microorganisms have been reported when these products reach their expiration date [26,27,28]. Higher TVC numbers were reported on the expiration date on both seasons for producer A, and high Enterobacteriaceae numbers were also reported for the same producer on the expiration date in winter. A study by Fröder et al. [45] revealed high Enterobacteriaceae and fecal coliforms populations (>2 log cfu/g) in different types of one leafy vegetable salads (iceberg lettuce, watercress, spinach, rocket, chicories) and mixed salads collected in spring and summer. High total mesophilic counts were also reported at the end of self-life of ready-to-eat rocket salads (lower than 7 log cfu/g) [46]. Summer was the season in which samples from producer A showed increased *Staphylococcus* spp. on their expiration date compared to the purchase date. On the other hand, samples from producer C presented low expiration *Staphylococcus* spp. numbers in summer. In our study, LAB counts were higher in winter for producer A on the product’s expiration date. Expiration date in summer presented higher yeasts and molds populations for producer B compared to winter. It is worth mentioning that according to De Corato [41], no significant variations of yeasts and molds counts were observed during the shelf life of the samples (rocket, baby leaf lettuce and lamb’s lettuce), while a significant variation on these populations and high numbers of fungi were evident only at the first day of shelf life. No significant differences were observed for coliforms, *E. coli* and *Pseudomonas* spp. in our study. The variation in the microbial load of ready-to-eat salads might be attributed to the possible different processing procedures applied by the producers/packagers [30].

Higher CO_2_ production and H_2_O_2_ levels were found in both seasons on the expiration date of samples from producers A and C. This might be attributed to tissue wounding (due to processing, mishandling) in combination with storage and display conditions (i.e., temperature, shelf life duration) that can accelerate the respiration rate of lettuce [47]. Lipid peroxidation and H_2_O_2_ levels were increased in winter on the last day of shelf life. Moreover, higher CO_2_ production was observed for producer E in winter and summer. Increased respiration rate for wild rocket salad was reported in spring to compare to summer (55.2 and 25.2 mL CO_2_/kg/h, respectively) when samples were stored at 5 °C and at the same time, rocket’s green color was preserved better in spring compared to summer [48]. However, it has been previously mentioned that lipid peroxidation resulting from plant stress (including increased respiration) can negatively affect the green color vegetables due to pigment bleaching (chlorophylls, carotenoids) and the production of brown pigments [49]. In our study, no differences were reported for phenols and antioxidants among seasons and days of analysis for all producers.

Expiration date in summer showed high TVC numbers for the combinations of lettuce with radicchio/endive, and rocket, while in winter, increased counts were found for the plain rocket, the combinations of lettuce with radicchio/endive and two/more ingredients (other). In a study by Sant’Ana et al. [50] in which the microbial load of nine different ready-to-eat vegetables (escarole, collard green, spinach, watercress, arugula, grated carrot, green salad, and mix for yakisoba) was assessed, it has been shown that total aerobic counts increased at the end of shelf life of the products (ranging from 2 to 8 log cfu/g) and this resulted from different storage temperatures (the higher the temperature, the higher the populations) as well as the type of vegetable among other factors [26]. Higher Enterobacteriaceae and coliform populations were found on the expiration date for the combination of lettuce and radicchio/endive on both seasons. Arvanitoyannis et al. [51] reported that a decrease in Enterobacteriaceae populations was evident (up to 0.5 log cfu/g) on the tenth day of storage with or without MAP. Interestingly in the same study, psychrotrophic counts were not influenced by the combination of lettuce with a rocket [51]. However, in our study, increased numbers of spoilage and psychrotrophic microorganisms (i.e., LAB, *Pseudomonas* spp., yeasts and molds) were observed on expiration date in summer for the combination of lettuce with rocket, as our ready-to-eat salads were stored in chilled conditions (7 °C). These observations might be due to improper handling and/or storage/transfer of ready-to-eat salads at inappropriate temperatures (up to 15 °C or even higher) in a commercial refrigerator. It is noteworthy that it has been previously mentioned that LAB have been isolated most from ready-to-eat vegetables under MAP [26,52]. Sant’Ana et al. [50] reported increased LAB populations on most ready-to-eat vegetables studied at the end of their shelf life when stored at abusive temperatures (15 °C). De Corato [41] reported that yeasts and mold counts were higher on the second day of shelf life for rocket salad on both seasons assessed (spring and summer) compared to lettuce and lamb’s lettuce salads. In our study, no significant differences were reported for *E. coli*, *Staphylococcus* spp. and *B. cereus* between purchase and expiration date among seasons for all types of salads.

Increased total phenolic content was reported on the expiration date of the combination of lettuce and chives in summer. On the other hand, decreased phenolics were reported for baby lettuce, curly endive and iceberg lettuce after 4 days of storage at 4 °C, while no significant differences among phenolic content were reported for radicchio, rocket and lamb’s lettuce [53]. This may be attributed to the packaging conditions in bagged samples due to the modified atmosphere packaging of these vegetables. Higher antioxidants (by FRAP assay) on product expiration date were observed in winter for the combination of lettuce with cabbage and in summer for the plain rocket. Preti and Vinci [53] reported increased antioxidants compounds (by DPPH assay) on the expiration date of baby lettuce, curly endive, lamb’s lettuce, rocket and radicchio salads. The majority of the combinations of lettuce with other ingredients showed higher H_2_O_2_ and MDA levels on the expiration date in both seasons. It is noteworthy to mention that Cavaiuolo et al. [27] reported a relation between lipid peroxidation and storage temperature of rocket, suggesting that storage of minimally processed vegetables, such as rocket at adverse (increased) temperatures increases respiration rate and negatively affects product quality due to plant stress and senescence. This is following our results since plain rocket showed higher CO_2_ production and MDA levels on the expiration date for both seasons. Arvanitoyannis et al. [51] reported increased CO_2_ levels of rocket with or without its combination with lettuce through storage at 5 °C for 10 days. Moreover, Nousiainen et al. [28] suggested that the increased CO_2_ production reported might have been attributed to the different types of vegetables as well as the microbial load of these products. These come following the findings of our study, where lettuce+endive in winter showed increased microbial load (TVC, Enterobacteriaceae and coliforms) and CO_2_ production on the expiration date. No significant differences were reported for antioxidant activity (by DPPH and ABTS assays) between purchase and expiration date among seasons for all types of salads.

## 5. Conclusions

The microbial load was varied, depending not only on the packager-salad producer but also on the mixtures of the different salad types. Therefore, common and safe sanitation management is important in the preparation of ready-to-eat salads. Summer was the season in which *Escherichia coli* counts were found to be higher for plain lettuce, but *Staphylococcus* spp. was increased in winter in plain rocket salads. Additionally. *Staphylococcus* spp. was increased in different salad-type mixtures, such as lettuce + endive/radicchio, lettuce + rocket and lettuce + chives in the summer period. *Listeria monocytogenes* were absent in any of the samples tested. Regarding expiration date (OR “estimated expiration date”), it was evident that microbial load (mainly spoilage microorganisms, such as *Pseudomonas* spp., yeasts and molds) increased during shelf life. Various salad types are respiring differently through the metabolic respiration process. The increased respiration rates through the increased CO_2_ production and damage indexes (H_2_O_2_ and MDA) observed on expiration date on both seasons indicating plant stress at the end of shelf life. These results suggest that the investigation of shelf life (from start to end) is essential for the understanding and development of novel technics monitoring the safety and quality of these products.

## Figures and Tables

**Table 1:** Effect of the sampling period (winter–summer) on microbiological load (log cfu/g), total phenolic content (mg GAE/g Fw), antioxidants (mg Trolox/g Fw), % CO_2_ and stress markers—H_2_O_2_ (μmol/g Fw) and MDA (nmol/g Fw) of ready-to-eat salads according to producer/packager (A, B, C, D and E).

	Producer/Packager				
	A	B	C	D	E
TVC	0.081	0.322	0.470	0.662	0.080
Enterobacteriaceae	0.750	0.706	0.858	0.492	0.608
Coliforms	0.105	0.280	0.080	0.527	0.829
*E. coli*	**0.022**	ni	**0.001**	0.485	ni
*Staphylococcus* spp.	0.399	0.937	0.114	0.285	ni
*B. cereus*	0.717	ni	**0.047**	ni	ni
Lactic acid bacteria	**0.000**	0.079	**0.000**	0.225	**0.010**
*Pseudomonas* spp.	0.100	**0.002**	0.568	0.107	0.817
Yeasts and molds	**0.002**	**0.033**	0.171	0.147	0.236
Phenols	0.786	0.327	**0.040**	0.853	0.479
DPPH	**0.000**	**0.001**	**0.000**	**0.017**	**0.000**
FRAP	**0.010**	0.277	0.093	0.324	**0.015**
ABTS	0.662	0.734	**0.020**	0.880	0.837
CO_2_	0.365	0.837	0.690	0.992	0.605
H_2_O_2_	0.708	0.877	0.297	0.838	0.284
MDA	**0.001**	0.871	0.436	0.139	0.930

Results shown are the *p* values following independent samples *t*-test, and bold values suggest significant differences (*p* < 5%). ni = the correlation and *t*-test could not be computed because the standard error of the difference was 0. A–E are salat producers. No additional info is necessary, as there are info at the M&M.

**Table 2:** Effect of the sampling period (winter–summer) on microbiological load (log cfu/g), total phenolic content (mg GAE/g Fw), antioxidants (mg Trolox/g Fw),% CO_2_ and stress markers—H_2_O_2_ (μmol/g Fw) and MDA (nmol/g Fw) of ready-to-eat salads according to the type of salad.

	Type of Salad						
	Lettuce	Lettuce + Cabbage	Lettuce + Endive/Radicchio	Lettuce + Rocket	Lettuce + Chives	Rocket	Other
TVC	0.131	0.244	0.373	0.949	0.827	0.343	0.511
Enterobacteriaceae	0.605	0.941	0.599	0.221	0.391	0.692	0.480
Coliforms	0.705	0.681	0.430	0.480	0.210	0.344	0.147
*E. coli*	**0.044**	0.168	0.336	0.408	0.178	0.082	0.432
*Staphylococcus* spp.	0.465	0.483	**0.016**	**0.012**	**0.028**	**0.032**	0.589
*B. cereus*	ni	ni	ni	0.645	ni	0.081	0.530
Lactic acid bacteria	**0.029**	**0.001**	**0.000**	**0.004**	**0.011**	**0.037**	**0.001**
*Pseudomonas* spp.	0.291	**0.035**	0.656	**0.003**	0.793	0.201	0.078
Yeasts and molds	0.254	0.092	**0.000**	**0.000**	0.887	0.089	**0.046**
Phenols	0.279	**0.026**	0.662	0.760	**0.005**	0.389	0.376
DPPH	**0.000**	**0.000**	**0.000**	**0.000**	**0.000**	**0.000**	**0.001**
FRAP	**0.002**	0.510	0.085	0.060	0.777	**0.004**	0.396
ABTS	0.230	0.177	0.353	0.589	**0.021**	0.184	0.320
CO_2_	0.771	0.989	0.464	0.745	0.179	0.955	0.897
H_2_O_2_	0.531	0.470	0.228	0.933	0.939	0.171	0.647
MDA	**0.002**	0.531	0.315	0.948	0.155	0.607	**0.003**

Results shown are the *p* values following independent samples *t*-test, and bold values suggest significant differences (*p* < 5%). Other = lettuce + 2 or more ingredients. ni = the correlation and *t*-test could not be computed because the standard error of the difference was 0.

**Table 3:** Effect of shelf life, salad producer/packager and type on microbiological load (log cfu/g), total phenolic content (mg GAE/g Fw), antioxidants (mg Trolox/g Fw) and% CO_2_ and stress markers—H_2_O_2_ (μmol/g Fw) and MDA (nmol/g Fw) of ready-to-eat salads according to salad producer in winter and summer.

	Producer/Packager									
	A		B		C		D		E	
	Winter	Summer	Winter	Summer	Winter	Summer	Winter	Summer	Winter	Summer
TVC	**0.003**	**0.036**	ni	0.119	0.691	0.303	0.058	0.837	0.953	0.777
Enterobacteriaceae	**0.025**	0.194	ni	0.391	0.418	0.702	0.397	0.892	0.522	0.153
Coliforms	0.061	0.432	ni	0.464	0.203	0.519	0.182	0.984	0.219	0.127
*E. coli*	ni	0.667	ni	ni	ni	0.506	0.391	0.500	ni	ni
*Staphylococcus* spp.	0.167	**0.014**	ni	0.500	0.443	0.732	0.927	0.500	ni	ni
*B. cereus*	0.329	0.339	ni	ni	ni	0.162	ni	ni	ni	ni
Lactic acid bacteria	**0.007**	0.574	ni	0.313	0.999	0.359	0.394	0.086	0.813	0.956
*Pseudomonas* spp.	0.692	0.237	ni	0.833	0.077	0.204	0.576	0.181	0.121	0.632
Yeasts and molds	0.682	0.093	ni	**0.045**	0.451	0.490	0.068	0.864	0.496	0.300
Phenols	0.062	0.665	ni	0.055	0.868	0.752	0.687	0.585	0.123	0.759
DPPH	0.446	0.444	ni	0.310	0.459	0.619	0.462	0.486	0.105	0.798
FRAP	0.203	0.312	ni	0.607	0.654	0.750	0.283	0.571	0.358	0.516
ABTS	0.091	0.952	ni	0.059	0.904	0.975	0.726	0.420	0.328	0.691
CO_2_	**0.000**	**0.000**	ni	0.226	**0.016**	**0.000**	0.117	0.525	**0.018**	**0.048**
H_2_O_2_	**0.000**	**0.000**	ni	0.407	**0.000**	**0.000**	**0.011**	0.09	**0.044**	0.155
MDA	**0.000**	**0.001**	ni	0.366	**0.000**	**0.000**	**0.047**	0.224	**0.009**	0.204

Results shown are the *p* values following independent samples *t*-test, and bold values suggest significant differences (*p* < 5%). Other = lettuce + 2 or more ingredients. ni = the correlation and *t*-test could not be computed because the standard error of the difference was 0.

**Table 4:** Effect of shelf life on microbiological load (log cfu/g), total phenolic content (mg GAE/g Fw), antioxidants (mg Trolox/g Fw), % CO_2_ and stress markers—H_2_O_2_ (μmol/g Fw) and MDA (nmol/g Fw) of ready-to-eat salads according to the type of salad in winter and summer.

	Type of Salad													
	Lettuce		Lettuce + Cabbage		Lettuce + Endive/Radicchio		Lettuce + Rocket		Lettuce + Chives		Rocket		Other	
	Winter	Summer	Winter	Summer	Winter	Summer	Winter	Summer	Winter	Summer	Winter	Summer	Winter	Summer
TVC	0.386	0.898	0.361	0.263	**0.029**	**0.027**	ni	**0.012**	0.294	0.384	**0.036**	0.810	**0.019**	0.943
Enterobacteriaceae	0.244	0.313	0.516	0.366	**0.011**	**0.024**	ni	0.206	0.286	0.389	0.482	0.999	0.121	0.871
Coliforms	**0.039**	0.145	0.686	0.364	**0.001**	0.276	ni	0.261	0.130	0.453	0.500	0.602	0.146	0.189
*E. coli*	ni	0.181	ni	0.178	ni	0.356	ni	0.437	ni	0.423	ni	0.648	0.343	0.391
*Staphylococcus* spp.	0.647	0.213	0.423	0.698	0.356	0.065	ni	0.667	ni	0.186	0.363	0.632	0.686	0.391
*B. cereus*	ni	ni	ni	ni	ni	ni	ni	0.391	ni	ni	ni	0.348	0.343	0.391
Lactic acid bacteria	0.197	0.784	0.307	0.551	0.105	0.206	ni	**0.025**	0.051	0.469	0.223	0.985	0.060	0.391
*Pseudomonas* spp.	0.880	0.397	0.423	0.778	0.298	0.135	ni	**0.040**	0.551	0.089	0.856	0.220	0.633	0.547
Yeasts and molds	0.188	0.159	0.406	0.180	0.329	0.309	ni	**0.001**	0.855	0.705	0.306	0.167	0.685	0.952
Phenols	0.982	0.496	0.487	0.884	0.168	0.988	ni	0.796	0.164	**0.010**	0.768	0.342	0.174	0.207
DPPH	0.797	0.419	0.161	0.512	0.234	0.583	ni	0.466	0.071	0.241	0.978	0.925	0.759	0.243
FRAP	0.821	0.355	**0.013**	0.843	0.384	0.696	ni	0.256	0.227	0.149	0.497	**0.044**	0.271	0.223
ABTS	0.883	0.725	0.059	0.563	0.293	0.660	ni	0.387	0.092	0.101	0.085	0.534	0.059	0.371
CO_2_	**0.000**	**0.014**	0.192	0.098	**0.017**	**0.016**	ni	**0.016**	0.173	**0.035**	**0.004**	0.085	**0.001**	0.050
H_2_O_2_	**0.000**	**0.002**	**0.005**	**0.016**	**0.000**	**0.040**	ni	0.128	0.193	**0.014**	**0.035**	**0.003**	**0.000**	**0.020**
MDA	**0.000**	**0.000**	**0.029**	**0.001**	**0.000**	**0.023**	ni	0.069	0.314	0.105	**0.004**	**0.006**	**0.000**	**0.034**

Results shown are the *p* values following paired samples *t*-test, and bold values suggest significant differences (*p* < 5%). Other = lettuce + 2 or more ingredients. ni = the correlation and *t*-test could not be computed because the standard error of the difference was 0.

## Data Availability

The data presented in this study are available on request from the corresponding author.

## Supplementary Materials

The following are available online at https://www.mdpi.com/article/10.3390/foods10050941/s1, Figure S1: Effects of shelf life (days) on microbiological quality per salad producer in winter and summer. Figure S2: Effects of shelf life (days) on microbiological quality per type of salad in winter and summer. Figure S3: Effects of shelf life (days) on total phenolic content, antioxidants, % CO_2_ and damage index (H_2_O_2_ and lipid peroxidation) per salad producer in winter and summer. Figure S4: Effects of shelf life (days) on total phenolic content, antioxidants, % CO_2_ and damage index (H_2_O_2_ and lipid peroxidation) per type of salad in winter and summer.
