# Supplementary material for: Expiration Date of Ready-to-Eat Salads: Effects on Microbial Load and Biochemical Attributes
Source: Foods. 2021 Apr 25;10(5):941. doi: 10.3390/foods10050941 (PMC8145991; doi:10.3390/foods10050941)
Supplement: Supplementary file 1 [file foods-10-00941-s001.zip › foods-1177997-supplementary.pdf]

Supplementary materials

	A	B	C	D	E
TVC	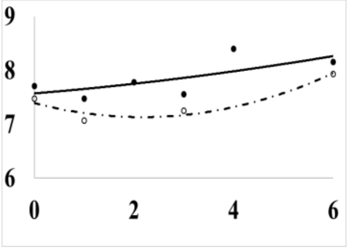	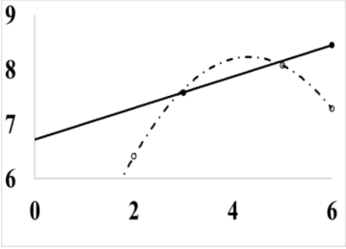	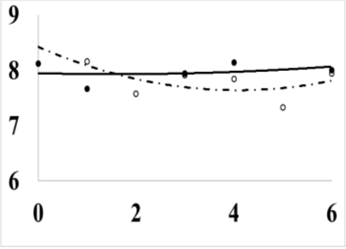	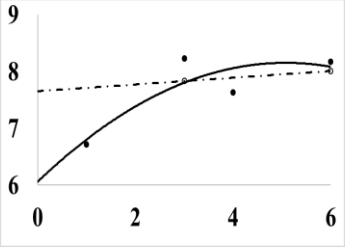	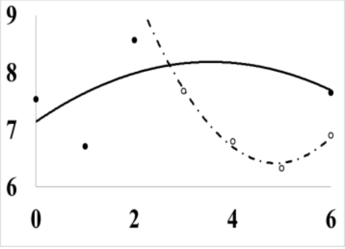
	$y=0.0065x^2+0.077x+7.5665$; $R^2=0.49$ (W)<br>$y=0.0556x^2-0.2409x+7.3881$; $R^2=0.92$ (S)	$y=0.2885x+6.7165$; $R^2=1$. (W)<br>$y=-0.338x^2+2.9236x+1.9116$; $R^2=1$ (S)	$y=0.007x^2-0.0219x+7.9462$; $R^2=0.08$ (W)<br>$y=0.0471x^2-0.3843x+8.4229$; $R^2=0.31$ (S)	$y=0.0594x+7.6531$; $R^2=1$ (W)<br>$y=-0.0813x^2+0.8249x+6.0599$; $R^2=0.74$ (S)	$y=-0.0827x^2+0.5883x+7.139$; $R^2=0.21$ (W)<br>$y=0.3661x^2-3.5761x+15.146$; $R^2=0.98$ (S)
Enterobacteriaceae	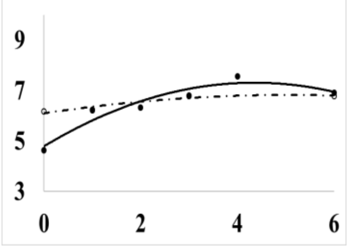	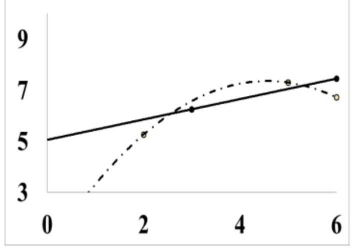	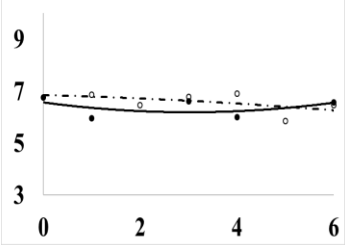	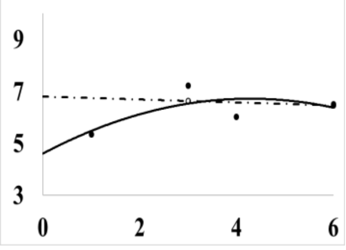	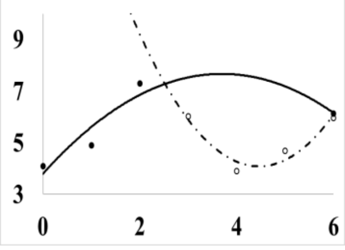
	$y=-0.1334x^2+1.1601x+4.78$; $R^2=0.92$ (W)<br>$y=-0.0284x^2+0.2881x+6.0894$; $R^2=0.90$ (S)	$y=-0.0284x^2+0.2881x+6.0894$; $R^2=0.90$ (W)<br>$y=-0.3162x^2+2.8925x+0.7456$; $R^2=1$ (S)	$y=0.0415x^2-0.2506x+6.5642$; $R^2=0.22$ (W)<br>$y=-0.0084x^2-0.0476x+6.8476$; $R^2=0.26$ (S)	$y=-0.1155x^2+0.9882x+4.6027$; $R^2=0.48$ (W)<br>$y=-0.0566x+6.8005$; $R^2=1$ (S)	$y=-0.2839x^2+2.0972x+3.7943$; $R^2=0.87$ (W)<br>$y=0.8475x^2-7.5607x+20.945$; $R^2=0.91$ (S)
Coliforms	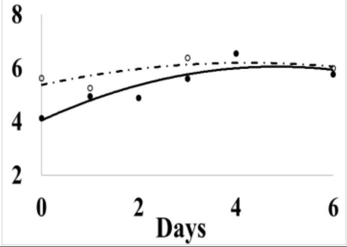	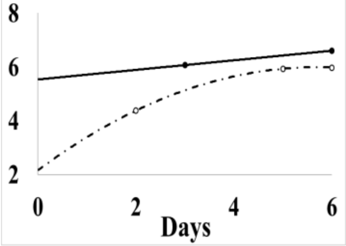	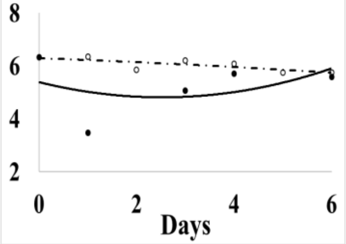	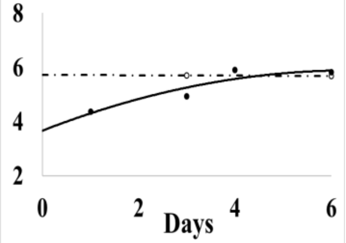	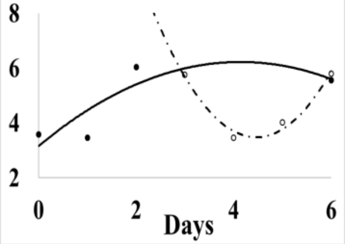
	$y=-0.0858x^2+0.8292x+4.04$; $R^2=0.83$ (W)<br>$y=-0.0464x^2+0.3923x+5.3613$; $R^2=0.50$ (S)	$y=0.1781x+5.5346$; $R^2 = 1$ (W)<br>$y=-0.1178x^2+1.3415x+2.1691$; $R^2=1$ (S)	$y=0.0903x^2-0.4554x+5.3894$; $R^2=0.16$ (W)<br>$y=-0.004x^2-0.068x+6.2958$; $R^2=0.51$ (S)	$y=-0.0537x^2+0.6912x+3.6615$; $R^2=0.86$ (W)<br>$y=-0.007x+5.7183$; $R^2=1$ (S)	$y=-0.1788x^2+1.4789x+3.1619$; $R^2=0.70$ (W)<br>$y=1.0244x^2-9.1515x+23.916$; $R^2=0.97$ (S)

Figure S1. Effects of shelf life (days) on microbiological quality (log cfu/g) per salad producer during winter (●, W) and summer (○, S). A: producer A, B: producer B, C: producer C, D: producer D, E: producer E.

	A	B	C	D	E
E. coli	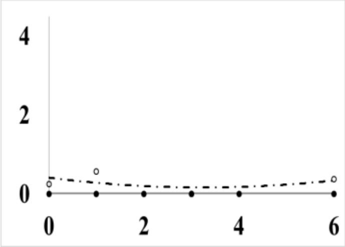	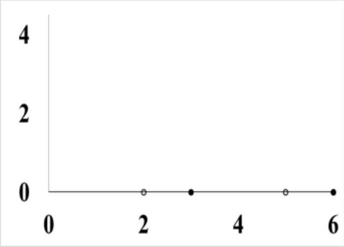	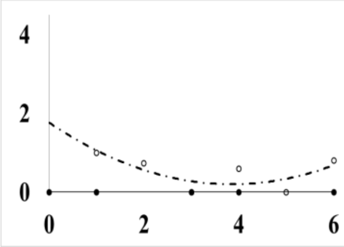	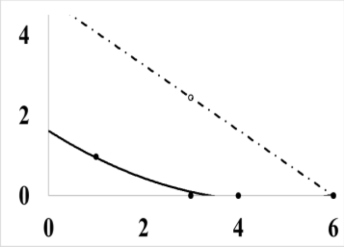	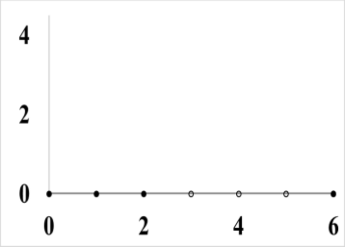
	$y=0.0234x^2-0.1522x+0.4085$; $R^2=0.19$ (W)<br>$y=0$; R^2=#N/A (S)	$y=0$; R^2=#N/A (W)<br>$y=0$; R^2=#N/A (S)	$y=0$; R^2=#N/A (W)<br>$y=0.1055x^2-0.8132x+1.7684$; $R^2=0.57$ (S)	$y=0.0804x^2-0.748x+1.6134$; $R^2=0.97$ (W)<br>$y=-0.8138x+4.8829$; $R^2=1$ (S)	$y=0$; R^2=#N/A (W)<br>$y=0$; R^2=#N/A (S)
Staphylococcus spp.	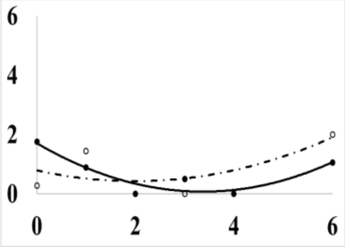	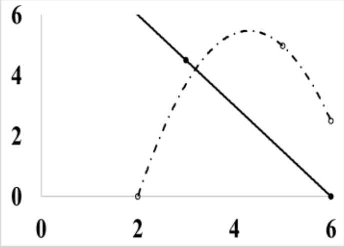	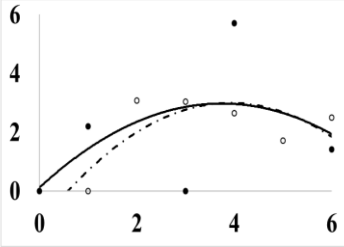	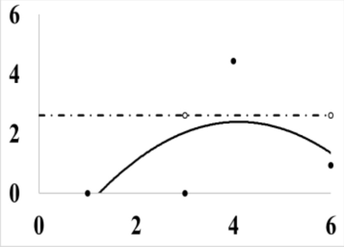	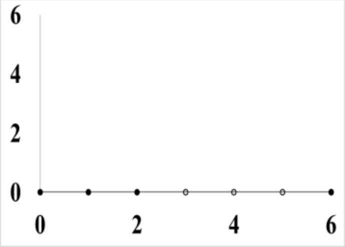
	$y=0.1439x^2-0.9699x+1.7015$; $R^2=0.87$ (W)<br>$y=0.092x^2-0.3665x+0.7854$; $R^2=0.48$ (S)	$y=-1.5004x+9.0026$; $R^2=1$ (W)<br>$y=-1.033x^2+8.8877x-13.643$; $R^2=1$ (S)	$y=-0.2039x^2+1.526x+0.1156$; $R^2=0.25$ (W)<br>$y=-0.2684x^2+2.1064x-1.141$; $R^2=0.53$ (S)	$y=-0.2909x^2+2.3877x-2.5049$; $R^2=0.35$ (W)<br>$y=0.0012x+2.6067$; $R^2=1$ (S)	$y=0$; R^2=#N/A (W)<br>$y=0$; R^2=#N/A (S)
B. cereus	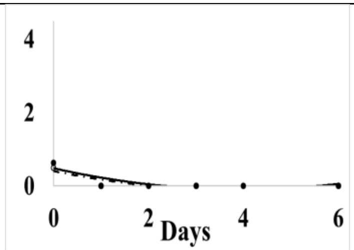	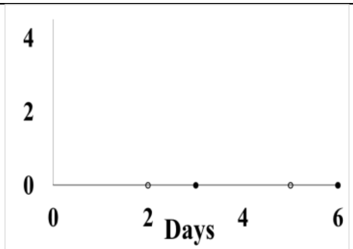	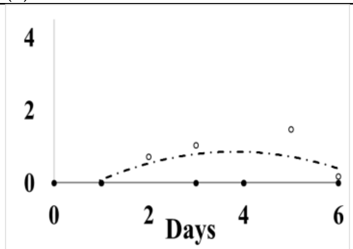	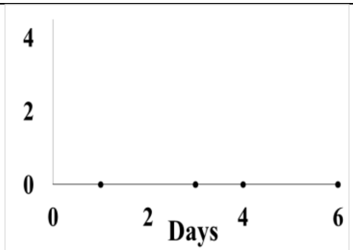	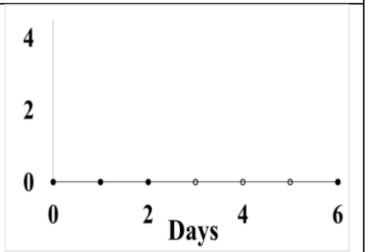
	$y=0.037x^2-0.2927x+0.4775$; $R^2=0.72$ (W)<br>$y=0.0336x^2-0.2648x+0.3991$; $R^2=0.75$ (S)	$y=0$; R^2=#N/A (W)<br>$y=0$; R^2=#N/A (S)	$y=0$; R^2=#N/A (W)<br>$y=-0.0975x^2+0.7434x-0.5562$; $R^2=0.22$ (S)	$y=0$; R^2=#N/A (W)<br>$y=0$; R^2=#N/A (S)	$y=0$; R^2=#N/A (W)<br>$y=0$; R^2=#N/A (S)

Figure S1. (Continued)

	A	B	C	D	E
**LAB**	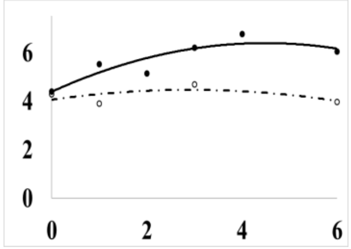	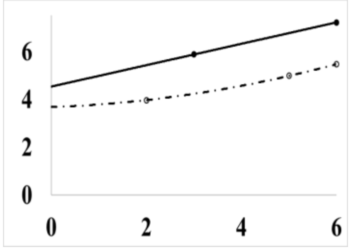	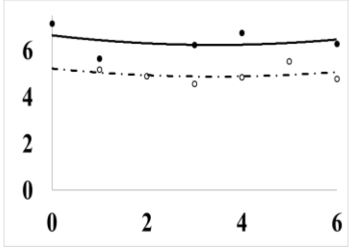	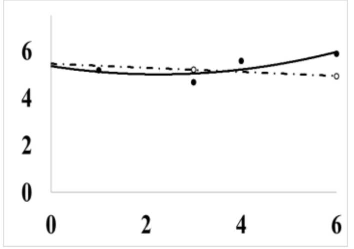	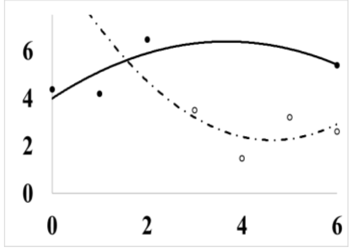
	$y=-0.0995x^2+0.8922x+4.387$; $R^2=0.80$ (W)<br>$y=-0.0481x^2+0.2799x+4.0557$; $R^2=0.36$ (S)	$y=0.4472x+4.5283$; $R^2=1$ (W)<br>$y=0.0382x^2+0.0692x+3.6735$; $R^2=1$ (S)	$y=0.0353x^2-0.2415x+6.6444$; $R^2=0.09$ (W)<br>$y=0.0292x^2-0.1991x+5.2173$; $R^2=0.06$ (S)	$y=0.0683x^2-0.3092x+5.351$; $R^2=0.66$ (W)<br>$y=-0.0861x+5.4525$; $R^2=1$ (S)	$y=-0.1775x^2+1.3026x+3.996$; $R^2=0.59$ (W)<br>$y=0.3588x^2-3.3222x+9.9299$; $R^2=0.24$ (S)
***Pseudomonas* spp.**	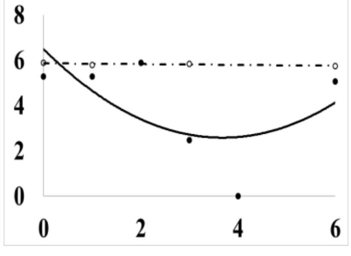	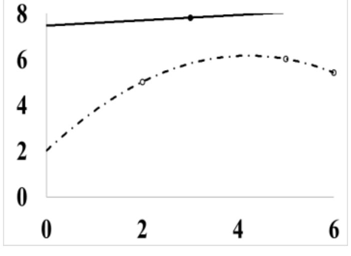	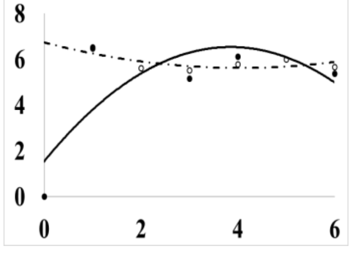	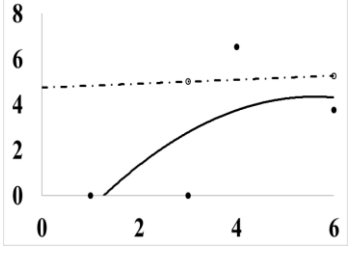	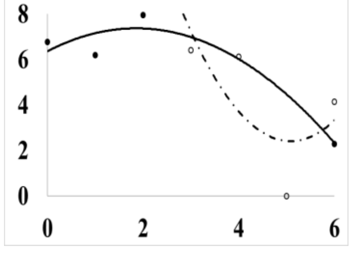
	$y=0.2886x^2-2.1231x+6.4817$; $R^2=0.40$ (W)<br>$y=-0.0005x^2-0.0145x+5.8552$; $R^2=0.49$ (S)	$y=0.1158x+7.4753$; $R^2=1$ (W)<br>$y=-0.2333x^2+1.967x+2.0187$; $R^2=1$ (S)	$y=-0.3361x^2+2.5894x+1.5497$; $R^2=0.59$ (W)<br>$y=0.0677x^2-0.5478x+6.7348$; $R^2=0.45$ (S)	$y=-0.2313x^2+2.5916x-2.9158$; $R^2=0.47$ (W)<br>$y=0.0873x+4.7441$; $R^2=1$ (S)	$y=-0.2927x^2+1.0838x+6.3571$; $R^2=0.92$ (W)<br>$y=1.1091x^2-11.276x+31.06$; $R^2=0.51$ (S)
**Yeasts and molds**	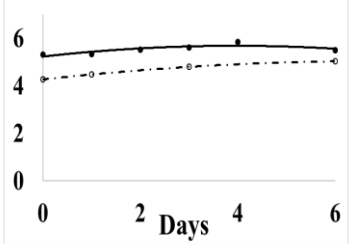	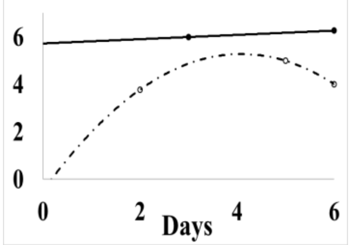	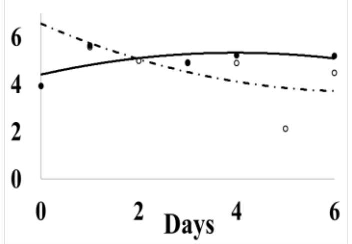	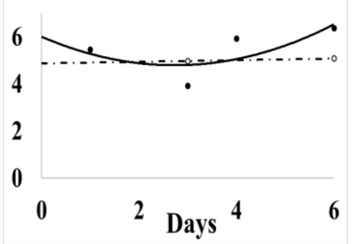	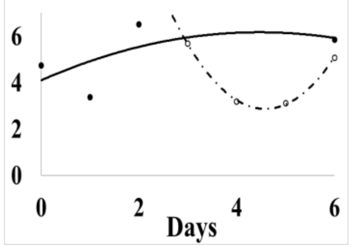
	$y=-0.0292x^2+0.2302x+5.2213$; $R^2=0.75$ (W)<br>$y=-0.0164x^2+0.2254x+4.261$; $R^2=1$ (S)	$y=0.0905x+5.7197$; $R^2=1$ (W)<br>$y=-0.3454x^2+2.8219x-0.4864$; $R^2=1$ (S)	$y=-0.0585x^2+0.4646x+4.4078$; $R^2=0.35$ (W)<br>$y=0.0692x^2-0.8898x+6.5646$; $R^2=0.41$ (S)	$y=0.1622x^2-0.8851x+5.9907$; $R^2=0.52$ (W)<br>$y=0.0346x+4.862$; $R^2=1$ (S)	$y=-0.104x^2+0.9281x+4.0994$; $R^2=0.34$ (W)<br>$y=1.1139x^2-10.212x+26.267$; $R^2=1$ (S)

Figure S1. (Continued)

	TVC		*Enterobacteriaceae*	
**Lettuce**	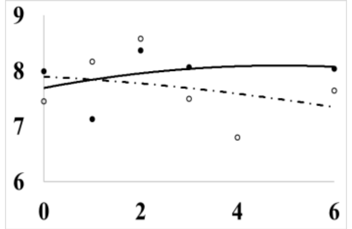	$y=-0.0171x^2+0.1662x+7.6891$; $R^2=0.11$ (W) $y=-0.0076x^2-0.0462x+7.8942$; $R^2=0.11$ (S)	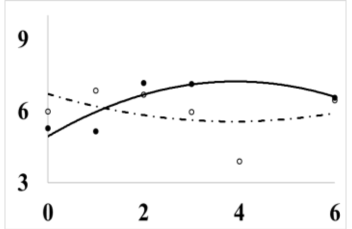	$y=-0.1492x^2+1.1711x+4.9355$; $R^2=0.74$ (W) $y=0.0773x^2-0.5979x+6.7124$; $R^2=0.16$ (S)
**Lettuce+Cabbage**	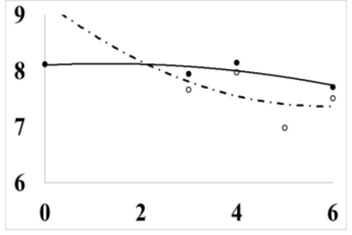	$y=-0.0173x^2+0.044x+8.0999$; $R^2=0.68$ (W) $y=0.0551x^2-0.6416x+9.2347$; $R^2=0.23$ (S)	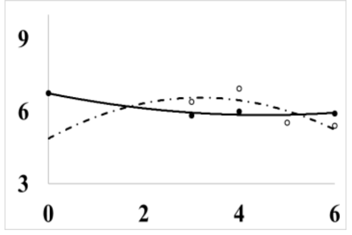	$y=0.0443x^2-0.4x+6.7485$; $R^2=0.94$ (W) $y=-0.1674x^2+1.0666x+4.8716$; $R^2=0.67$ (S)
**Lettuce+Endive/ radicchio**	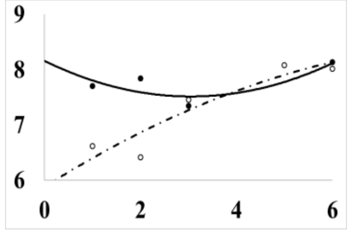	$y=0.0694x^2-0.4229x+8.156$; $R^2=0.68$ (W) $y=-0.0287x^2+0.5487x+5.8757$; $R^2=0.86$ (S)	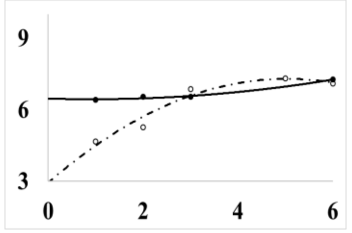	$y=0.032x^2-0.0589x+6.4607$; $R^2=0.99$ (W) $y=-0.1721x^2+1.7325x+2.9305$; $R^2=0.95$ (S)
**Lettuce+Rocket**	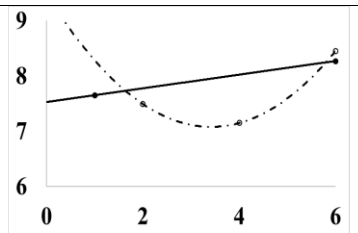	$y=0.1238x+7.5249$; $R^2=1$ (W) $y=0.2062x^2-1.4092x+9.4822$; $R^2=1$ (S)	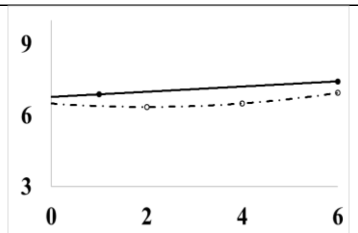	$y=0.1095x+6.7699$; $R^2=1$ (W) $y=0.0369x^2-0.147x+6.4949$; $R^2=1$ (S)
**Lettuce+Chives**	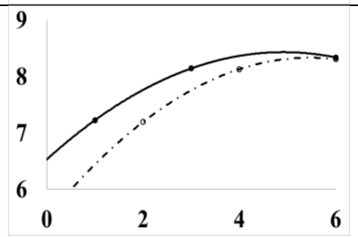	$y=-0.0787x^2+0.7739x+6.528$; $R^2=1$ (W) $y=-0.0951x^2+1.0385x+5.4959$; $R^2=1$ (S)	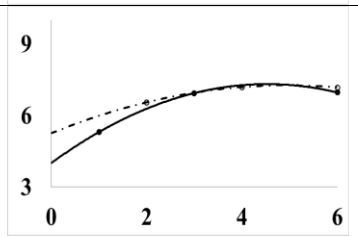	$y=-0.162x^2+1.4662x+3.9996$; $R^2=1$ (W) $y=-0.0821x^2+0.813x+5.2556$; $R^2=1$ (S)
**Rocket**	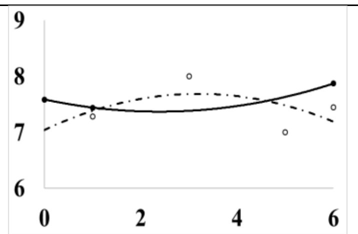	$y=0.0384x^2-0.1831x+7.5846$; $R^2=1$ (W) $y=-0.0633x^2+0.4044x+7.0396$; $R^2=0.24$ (S)	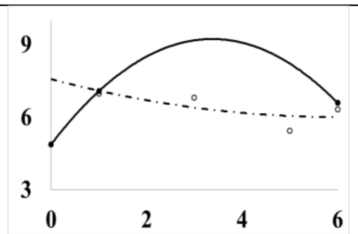	$y=-0.3829x^2+2.5829x+4.8686$; $R^2=1$ (W) $y=0.0438x^2-0.5241x+7.567$; $R^2=0.54$ (S)
**Other**	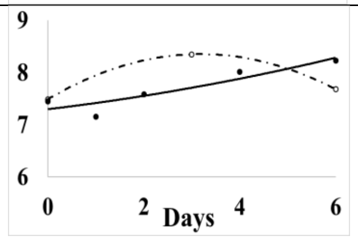	$y=0.0091x^2+0.1099x+7.2934$; $R^2=0.85$ (W) $y=-0.0851x^2+0.542x+7.487$; $R^2=1$ (S)	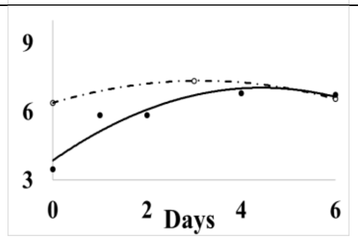	$y=-0.1626x^2+1.4405x+3.8483$; $R^2=0.89$ (W) $y=-0.0982x^2+0.6176x+6.3752$; $R^2=1$ (S)

Figure S2. Effects of shelf life (days) on microbiological quality (log cfu/g) per type of salad during winter (●, W) and summer (○, S).Other= Lettuce +2 or more ingredients.

	Coliforms		*E. coli*	
**Lettuce**	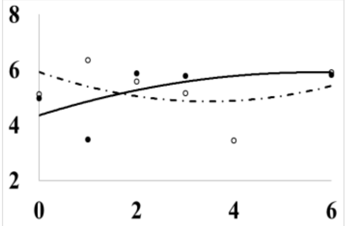	$y=-0.0488x^2+0.5507x+4.3534$; $R^2=0.35$ (W)<br>$y=0.0887x^2-0.616x+5.9244$; $R^2=0.17$ (S)	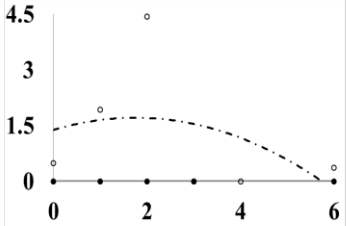	$y=0$; R^2=#N/A (W)<br>$y=-0.1072x^2+0.375x+1.3835$; $R^2=0.18$ (S)
**Lettuce+Cabbage**	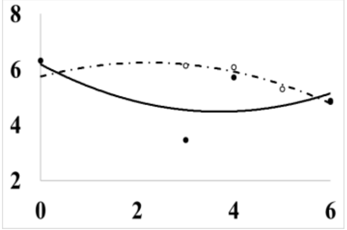	$y=0.1246x^2-0.9223x+6.1855$; $R^2=0.40$ (W)<br>$y=-0.1024x^2+0.4542x+5.7427$; $R^2=0.96$ (S)	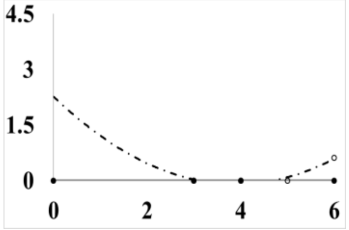	$y=0$; R^2=#N/A (W)<br>$y=0.1552x^2-1.2109x+2.2665$; $R^2=0.93$ (S)
**Lettuce+Endive/ radicchio**	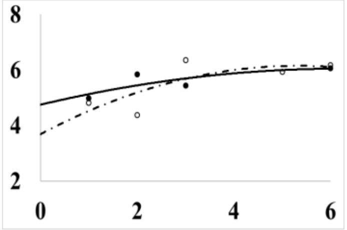	$y=-0.033x^2+0.4141x+4.746$; $R^2=0.65$ (W)<br>$y=-0.0866x^2+0.9227x+3.688$; $R^2=0.60$ (S)	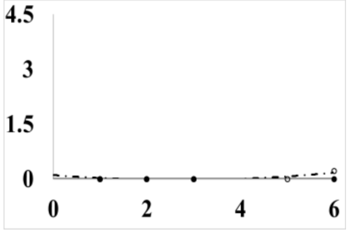	$y=0$; R^2=#N/A (W)<br>$y=0.0188x^2-0.1017x+0.1055$; $R^2=0.78$ (S)
**Lettuce+Rocket**	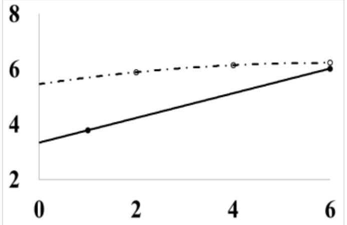	$y=0.4471x+3.3337$; $R^2=1$ (W)<br>$y=-0.022x^2+0.26x+5.4533$; $R^2=1$ (S)	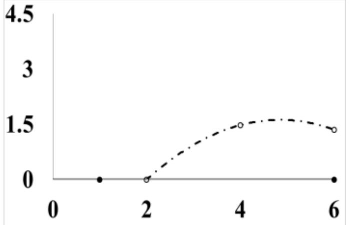	$y=0$; R^2=#N/A (W)<br>$y=-0.2003x^2+1.9402x-3.0792$; $R^2=1$ (S)
**Lettuce+Chives**	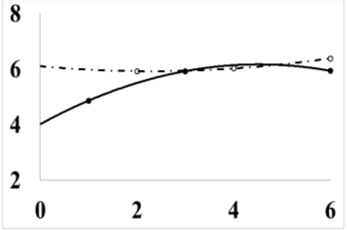	$y=-0.1053x^2+0.9517x+4.0176$; $R^2=1$ (W)<br>$y=0.0337x^2-0.1546x+6.1008$; $R^2=1$ (S)	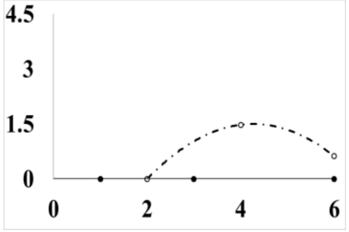	$y=0$; R^2=#N/A (W)<br>$y=-0.2915x^2+2.4877x-3.8093$; $R^2=1$ (S)
**Rocket**	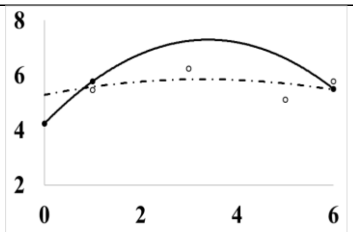	$y=-0.2648x^2+1.7995x+4.2344$; $R^2=1$ (W)<br>$y=-0.0528x^2+0.3472x+5.2809$; $R^2=0.11$ (S)	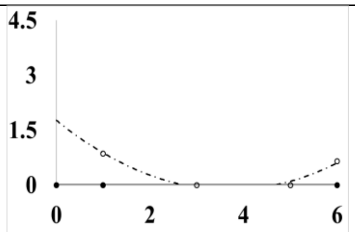	$y=0$; R^2=#N/A (W)<br>$y=0.1387x^2-1.0267x+1.7657$; $R^2=0.97$ (S)
**Other**	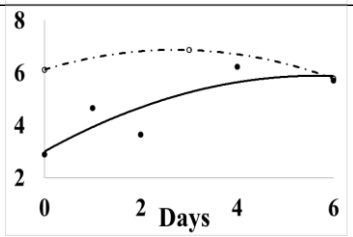	$y=-0.0902x^2+1.0194x+3.0019$; $R^2=0.74$ (W)<br>$y=-0.1018x^2+0.5537x+6.1195$; $R^2=1$ (S)	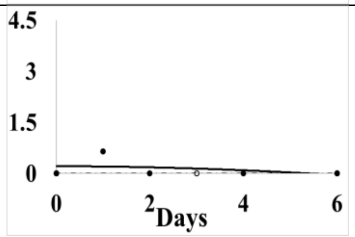	$y=-0.0069x^2-0.0027x+0.2138$; $R^2=0.15$ (W)<br>$y=0$; R^2=#N/A (S)

Figure S2. (Continued)

	Staphylococcus spp.		*B. cereus*	
**Lettuce**	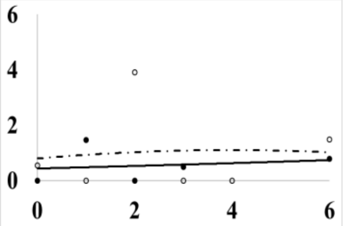	$y=0.001x^2+0.0445x+0.4313$; $R^2=0.04$ (W) $y=-0.0193x^2+0.1536x+0.7927$; $R^2=0.01$ (S)	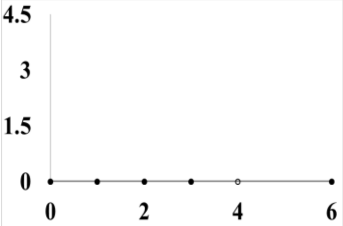	$y=0$; R^2=#N/A (W) $y=0$; R^2=#N/A (S)
**Lettuce+Cabbage**	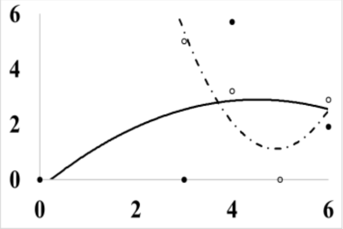	$y=-0.1574x^2+1.4217x-0.3165$; $R^2=0.31$ (W) $y=1.1715x^2-11.5x+29.333$; $R^2=0.78$ (S)	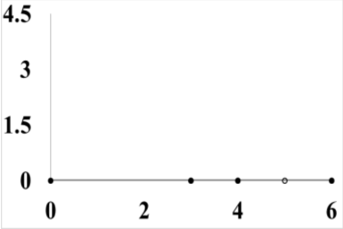	$y=0$; R^2=#N/A (W) $y=0$; R^2=#N/A (S)
**Lettuce+Endive/ radicchio**	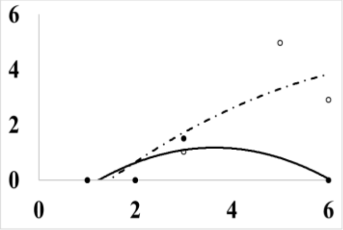	$y=-0.2031x^2+1.4797x-1.5253$; $R^2=0.63$ (W) $y=-0.0892x^2+1.5092x-2.013$; $R^2=0.73$ (S)	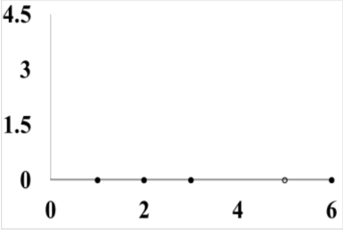	$y=0$; R^2=#N/A (W) $y=0$; R^2=#N/A (S)
**Lettuce+Rocket**	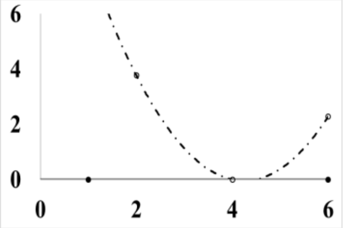	$y=0$; R^2=#N/A (W) $y=0.7558x^2-6.4166x+13.574$; $R^2=1$ (S)	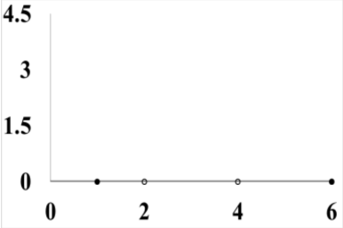	$y=0$; R^2=#N/A (W) $y=0$; R^2=#N/A (S)
**Lettuce+Chives**	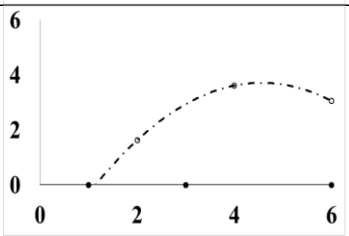	$y=0$; R^2=#N/A (W) $y=-0.3182x^2+2.9032x-2.9059$; $R^2=1$ (S)	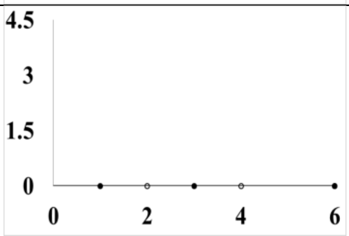	$y=0$; R^2=#N/A (W) $y=0$; R^2=#N/A (S)
**Rocket**	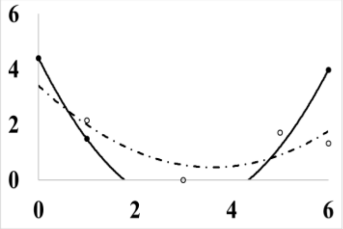	$y=0.5686x^2-3.483x+4.3915$; $R^2=1$ (W) $y=0.227x^2-1.6362x+3.4025$; $R^2=0.56$ (S)	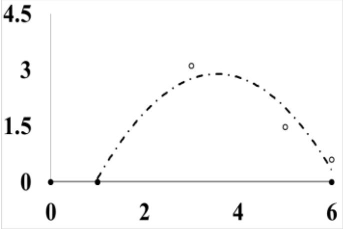	$y=0$; R^2 =#N/A (W) $y=-0.429x^2+3.0461x-2.5144$; $R^2=0.91$ (S)
**Other**	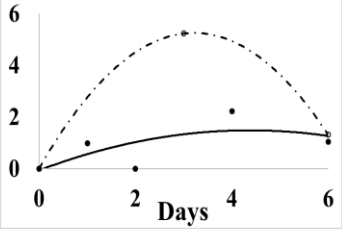	$y=-0.081x^2+0.7081x-0.0717$; $R^2=0.45$ (W) $y=-0.5075x^2+3.2626x+2E^{-15}$; $R^2=1$ (S)	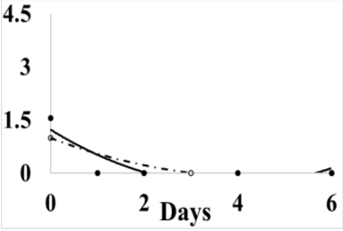	$y=0.1051x^2-0.8118x+1.223$; $R^2=0.73$ (W) $y=0.0547x^2-0.4924x+0.9847$; $R^2=1$ (S)

Figure S2. (Continued)

	LAB		*Pseudomonas* spp.	
**Lettuce**	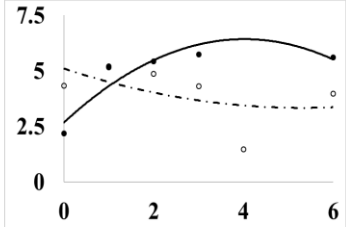	$y=-0.2322x^2+1.866x+2.6786$; $R^2=0.86$ (W)<br>$y=0.0623x^2-0.664x+5.1021$; $R^2=0.26$ (S)	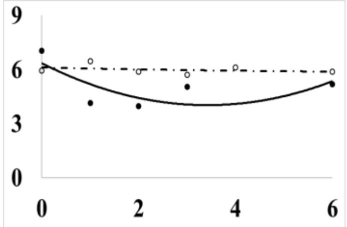	$y=0.1992x^2-1.3614x+6.349$; $R^2=0.53$ (W)<br>$y=0.0031x^2-0.0558x+6.1063$; $R^2=0.09$ (S)
**Lettuce+Cabbage**	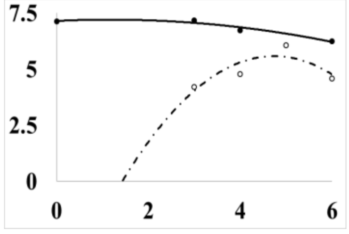	$y=-0.0424x^2+0.0989x+7.1523$; $R^2=0.94$ (W)<br>$y=-0.5107x^2+4.8365x-5.8641$; $R^2=0.69$ (S)	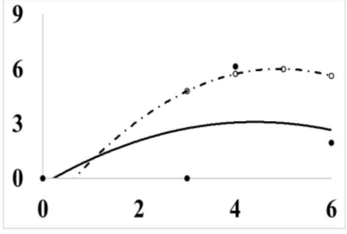	$y=-0.1758x^2+1.5512x-0.3412$; $R^2=0.30$ (W)<br>$y=-0.3288x^2+3.2399x-1.9806$; $R^2=1$ (S)
**Lettuce+Endive/ radicchio**	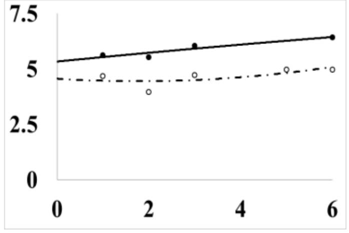	$y=-0.0033x^2+0.2039x+5.3372$; $R^2=0.87$ (W)<br>$y=0.0363x^2-0.1287x+4.5623$; $R^2=0.47$ (S)	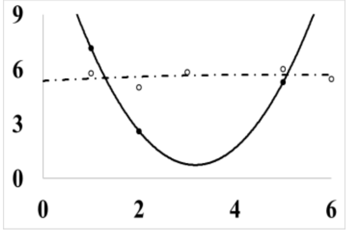	$y=1.3629x^2-8.6419x+14.44$; $R^2=1$ (W)<br>$y=-0.0151x^2+0.1444x+5.3624$; $R^2=0.05$ (S)
**Lettuce+Rocket**	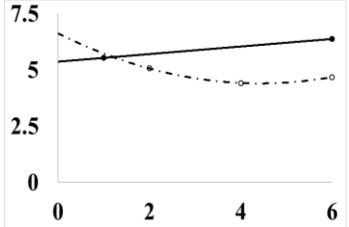	$y=0.1683x+5.3637$; $R^2=1$ (W)<br>$y=0.1141x^2-1.0079x+6.6222$; $R^2=1$ (S)	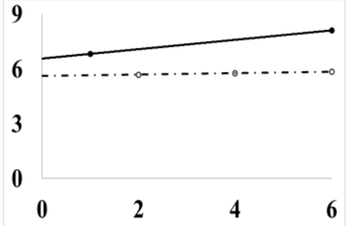	$y=0.257x+6.5674$; $R^2=1$ (W)<br>$y=0.0403x+5.6089$; $R^2=1$ (S)
**Lettuce+Chives**	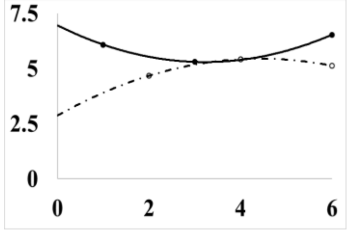	$y=0.1606x^2-1.0342x+6.9583$; $R^2=1$ (W)<br>$y=-0.1315x^2+1.1643x+2.8709$; $R^2=1$ (S)	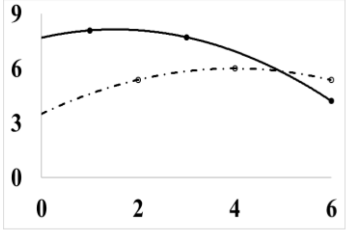	$y=-0.1964x^2+0.5984x+7.6855$; $R^2=1$ (W)<br>$y=-0.1568x^2+1.2492x+3.5068$; $R^2=1$ (S)
**Rocket**	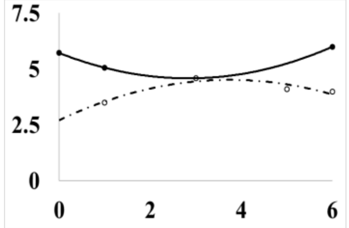	$y=0.1411x^2-0.8001x+5.7196$; $R^2=1$ (W)<br>$y=-0.1287x^2+0.9638x+2.7154$; $R^2=0.86$ (S)	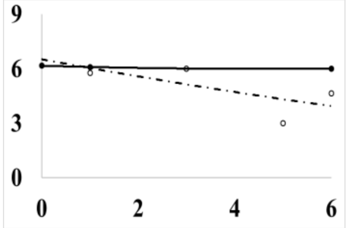	$y=0.0087x^2-0.0773x+6.1577$; $R^2=1$ (W)<br>$y=0.0101x^2-0.4904x+6.5128$; $R^2=0.46$ (S)
**Other**	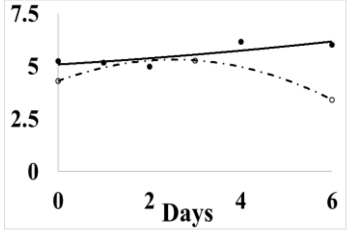	$y=0.0088x^2+0.1287x+5.0841$; $R^2=0.67$ (W)<br>$y=-0.1584x^2+0.8017x+4.2979$; $R^2=1$ (S)	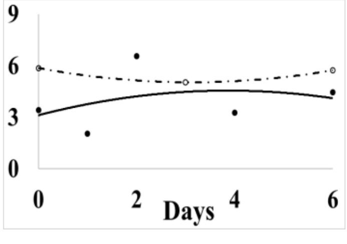	$y=-0.0961x^2+0.7417x+3.1075$; $R^2=0.10$ (W)<br>$y=0.0849x^2-0.531x+5.8563$; $R^2=1$ (S)

Figure S2. (Continued)

	Yeasts and molds	
Lettuce	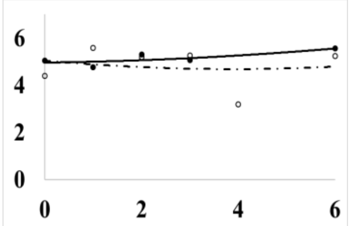	$y=0.0124x^2+0.0256x+4.9565$; $R^2=0.63$ (W) $y=0.0237x^2-0.1847x+5.0409$; $R^2=0.02$ (S)
Lettuce+Cabbage	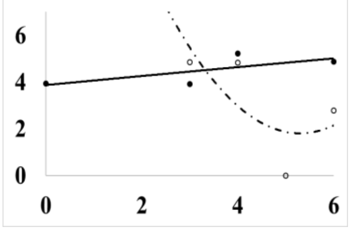	$y=-0.0007x^2+0.194x+3.8681$; $R^2=0.51$ (W) $y=0.6957x^2-7.3694x+21.318$; $R^2=0.51$ (S)
Lettuce+Endive/ radicchio	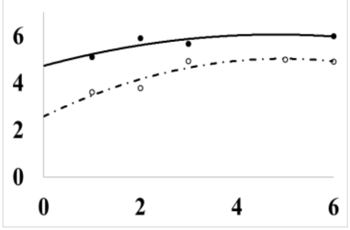	$y=-0.0577x^2+0.5543x+4.7211$; $R^2=0.70$ (W) $y=-0.1x^2+0.9909x+2.5704$; $R^2=0.87$ (S)
Lettuce+Rocket	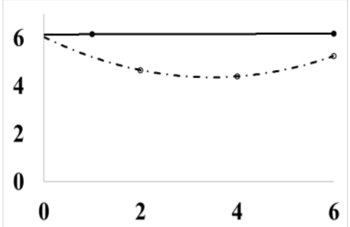	$y=0.0058x+6.1365$; $R^2=1$ (W) $y=0.1393x^2-0.9671x+6.028$; $R^2=1$ (S)
Lettuce+Chives	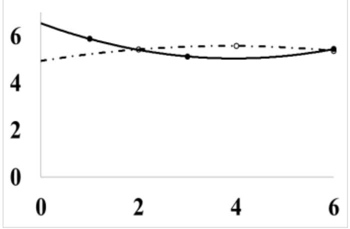	$y=0.0954x^2-0.754x+6.5341$; $R^2=1$ (W) $y=-0.0437x^2+0.3338x+4.9392$; $R^2=1$ (S)
Rocket	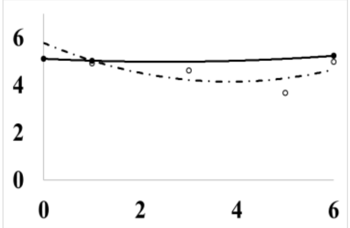	$y=0.0207x^2-0.104x+5.1068$; $R^2=1$ (W) $y=0.1108x^2-0.8521x+5.7727$; $R^2=0.39$ (S)
Other	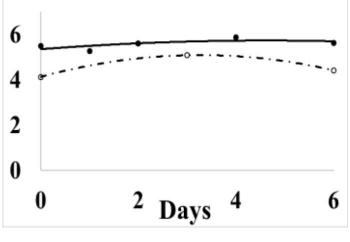	$y=-0.0179x^2+0.1653x+5.3446$; $R^2=0.47$ (W) $y=-0.0901x^2+0.5886x+4.1232$; $R^2=1$ (S)

Figure S2. (Continued)

	A	B	C	D	E
**Phenols (mg GAE/gFw)**	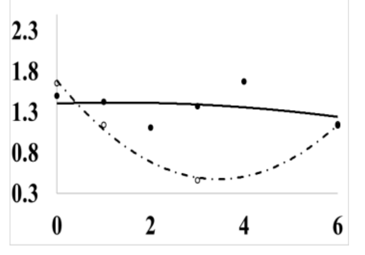	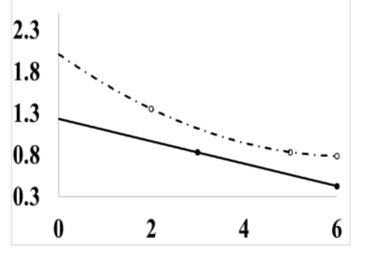	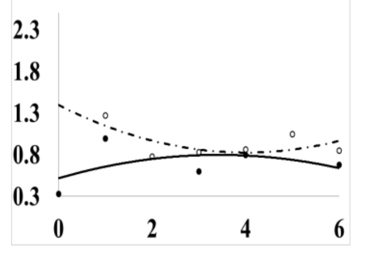	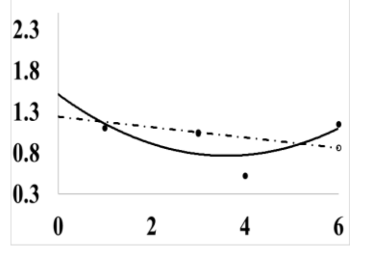	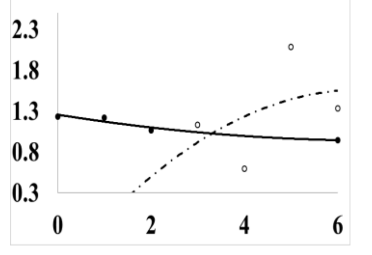
	$y=-0.0079x^2+0.0199x+1.402$; $R^2=0.10$ (W) $y=0.1022x^2-0.7038x+1.6862$; $R^2=0.99$ (S)	$y=-0.1344x+1.2311$; $R^2=1$ (W) $y=0.0313x^2-0.3917x+2.0093$; $R^2=1$ (S)	$y=-0.0239x^2+0.1638x+0.5166$; $R^2=0.22$ (W) $y=0.0356x^2-0.2852x+1.3969$; $R^2=0.40$ (S)	$y=0.0578x^2-0.4155x+1.5112$; $R^2=0.48$ (W) $y=-0.0631x+1.2387$; $R^2=1$ (S)	$y=0.0066x^2-0.0918x+1.2557$; $R^2=0.93$ (W) $y=-0.0525x^2+0.6828x-0.6584$; $R^2=0.20$ (S)
**DPPH (mg trolox/g Fw)**	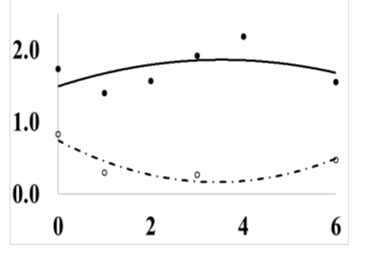	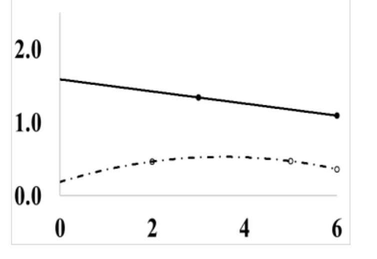	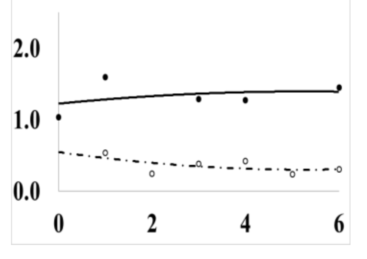	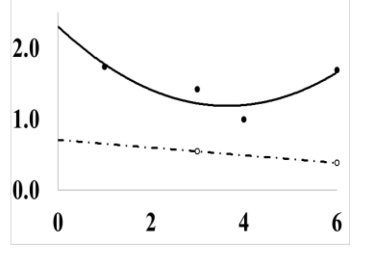	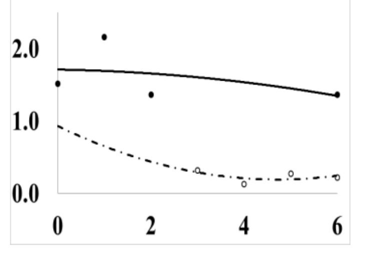
	$y=-0.0296x^2+0.209x+1.493$; $R^2=0.24$ (W) $y=0.0492x^2-0.3372x+0.7425$; $R^2=0.80$ (S)	$y=-0.0824x+1.5884$; $R^2=1$ (W) $y=-0.0274x^2+0.1933x+0.1901$; $R^2=1$ (S)	$y=-0.0062x^2+0.0648x+1.2247$; $R^2=0.12$ (W) $y=0.0089x^2-0.0935x+0.5475$; $R^2=0.30$ (S)	$y=0.0844x^2-0.6149x+2.3046$; $R^2=0.77$ (W) $y=-0.0539x+0.7051$; $R^2=1$ (S)	$y=-0.0084x^2-0.0104x+1.7113$; $R^2=0.20$ (W) $y=0.0331x^2-0.3127x+0.9319$; $R^2=0.28$ (S)
**FRAP (mg trolox.g Fw)**	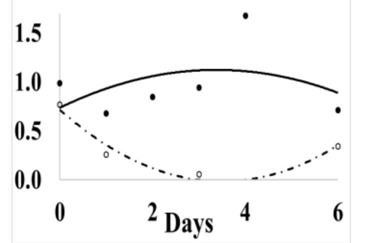	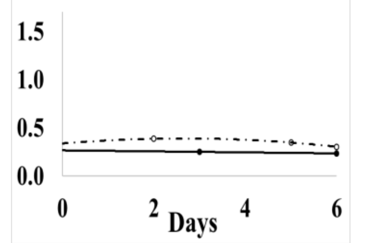	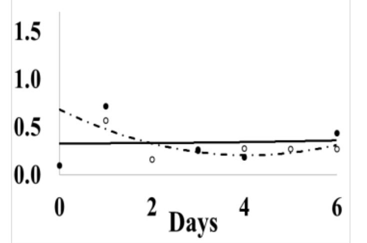	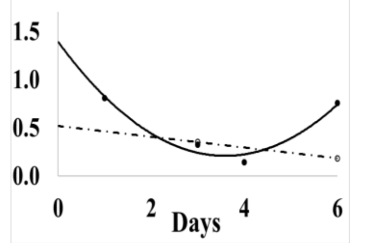	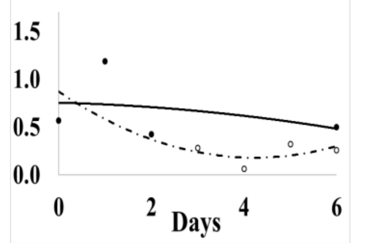
	$y=-0.0339x^2+0.2287x+0.7359$; $R^2=0.17$ (W) $y=0.0594x_2-0.4166x+0.7132$; $R^2=0.94$ (S)	$y=-0.0052x+0.2648$; $R^2=1$ (W) $y=-0.0076x^2+0.0399x+0.337$; $R^2=1$ (S)	$y=0.0009x^2-0.0006x+0.3275$; $R^2=0.01$ (W) $y=0.0289x^2-0.235x+0.6815$; $R^2=0.53$ (S)	$y=0.0919x^2-0.6597x+1.3911$; $R^2=0.96$ (W) $y=-0.0557x+0.5168$; $R^2=1$ (S)	$y=-0.0055x^2-0.012x+0.7524$; $R^2=0.13$ (W) $y=0.0387x^2-0.3268x+0.8693$; $R^2=0.20$ (S)

Figure S3. Effects of shelf life (days) on total phenolic content, antioxidants, % CO_2 and damage index (H_2O_2 and lipid peroxidation) per salad producer during winter (●, W) and summer (○, S). A: producer A, B: producer B, C: producer C, D: producer D, E: producer E.

	A	B	C	D	E
ABTS (mg trolox/gFw)	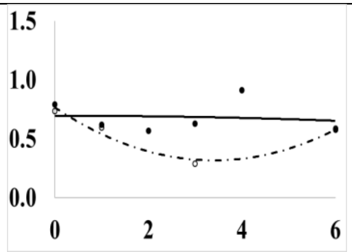	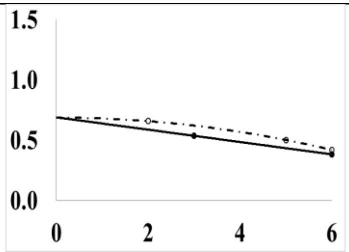	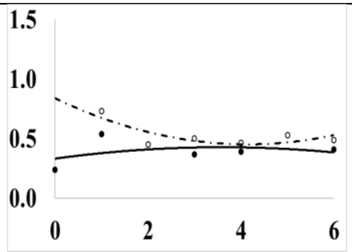	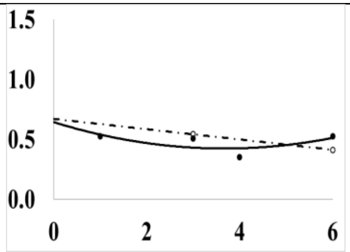	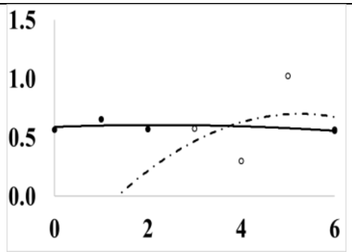
	$y=-0.0013x^2+0.0008x+0.6931$; $R^2=0.01$ (W) $y=0.0388x^2-0.264x+0.7642$; $R^2=0.95$ (S)	$y=-0.0512x+0.6899$; $R^2=1$ (W) $y=-0.0075x^2+0.0004x+0.6875$; $R^2=1$ (S)	$y=-0.0074x^2+0.0533x+0.3323$; $R^2=0.13$ (W) $y=0.0225x^2-0.1865x+0.8383$; $R^2=0.63$ (S)	$y=0.0164x^2-0.1201x+0.644$; $R^2=0.46$ (W) $y=-0.0429x+0.6709$; $R^2=1$ (S)	$y=-0.0034x^2+0.015x+0.5899$; $R^2=0.24$ (W) $y=-0.0459x^2+0.4818x-0.5632$; $R^2=0.12$ (S)
%CO_2	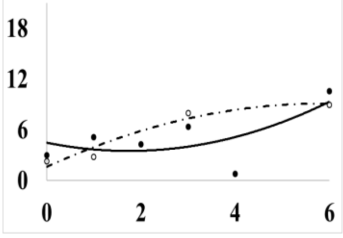	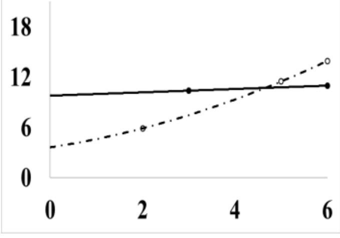	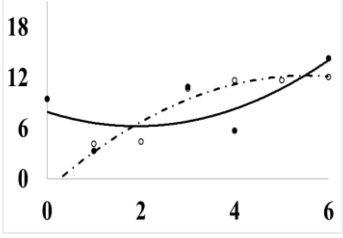	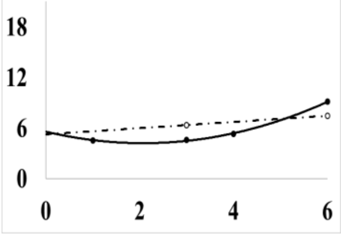	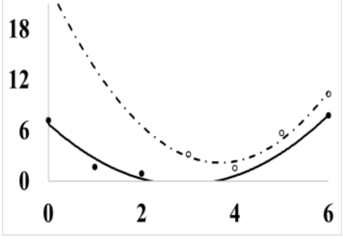
	$y=0.3216x^2-1.1189x+4.4455$; $R^2=0.44$ (W) $y=-0.2193x^2+2.5629x+1.5943$; $R^2=0.94$ (S)	$y=0.2x+9.77$; $R^2=1$ (W) $y=0.1458x^2+0.8458x+3.605$; $R^2=1$ (S)	$y=0.4674x^2-1.7828x+7.9173$; $R^2=0.50$ (W) $y=-0.4411x^2+4.8788x-1.2666$; $R^2=0.88$ (S)	$y=0.3179x^2-1.3187x+5.5714$; $R^2=0.99$ (W) $y=0.3683x+5.25$; $R^2=1$ (S)	$y=0.8554x^2-4.9645x+6.765$; $R^2=0.96$ (W) $y=1.56x^2-11.472x+23.244$; $R^2=0.97$ (S)
H_2O_2 (μmol/g Fw)	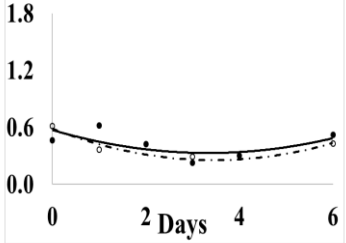	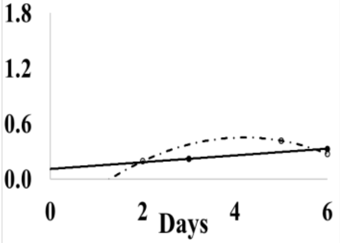	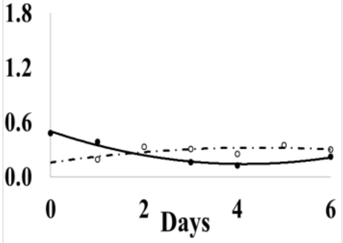	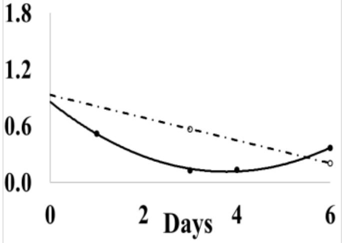	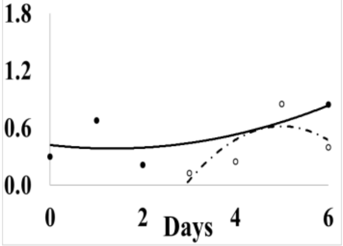
	$y=0.0219x^2-0.1457x+0.5722$; $R^2=0.44$ (W) $y=0.0277x^2-0.1913x+0.5848$; $R^2=0.92$ (S)	$y=0.0367x+0.1105$; $R^2=1$ (W) $y=-0.0536x^2+0.4491x-0.489$; $R^2=1$ (S)	$y=0.0209x^2-0.1735x+0.5001$; $R^2=0.98$ (W) $y=-0.0082x^2+0.0732x+0.1573$; $R^2=0.41$ (S)	$y=0.0519x^2-0.3925x+0.8563$; $R^2=0.99$ (W) $y=-0.1209x+0.9312$; $R^2=1$ (S)	$y=0.0207x^2-0.0554x+0.4204$; $R^2=0.52$ (W) $y=-0.1427x^2+1.4248x-2.9394$; $R^2=0.60$ (S)

Figure S3. (Continued)

	A	B	C	D	E
**MDA (nmol/gFw)**	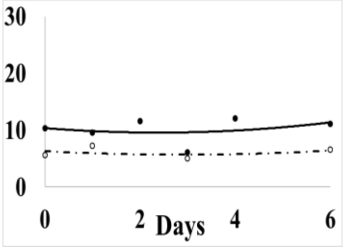	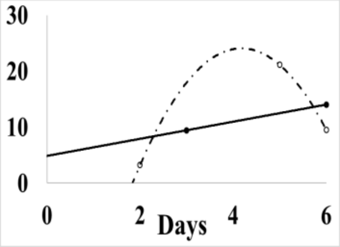	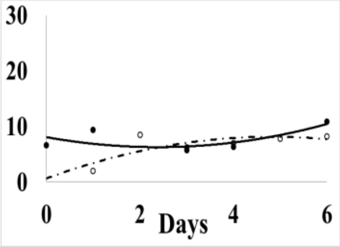	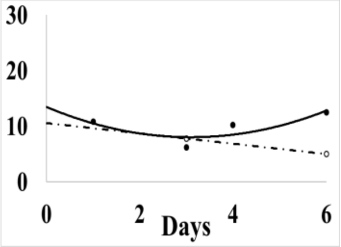	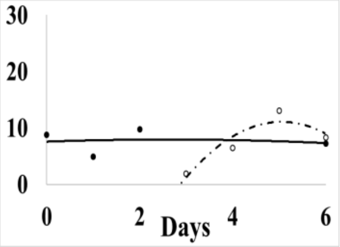
	$y=0.138x^2-0.6603x+10.357$; $R^2=0.10$ (W)<br>$y=0.0763x^2-0.4405x+6.3129$; $R^2=0.12$ (S)	$y=1.5271x+4.8657$; $R^2=1$ (W)<br>$y=-4.3854x^2+36.658x-52.53$; $R^2=1$ (S)	$y=0.3184x^2-1.5166x+8.0841$; $R^2=0.52$ (W)<br>$y=-0.3185x^2+3.0896x+0.661$; $R^2=0.58$ (S)	$y=0.5684x^2-3.5204x+13.487$; $R^2=0.68$ (W)<br>$y=-0.9297x+10.592$; $R^2=1$ (S)	$y=-0.0548x^2+0.2859x+7.5731$; $R^2=0.02$ (W)<br>$y=-2.3342x^2+23.588x-48.512$; $R^2=0.86$ (S)

Figure S3. (Continued)

	Phenols (mg GAE/g Fw)		DPPH (mg trolox/g Fw)	
**Lettuce**	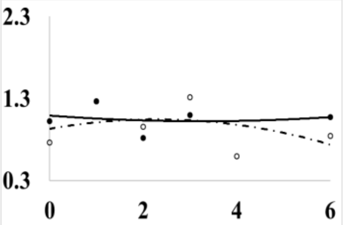	$y=0.0065x^2-0.0423x+1.0913$; $R^2=0.03$ (W)<br>$y=-0.0223x^2+0.102x+0.9298$; $R^2=0.16$ (S)	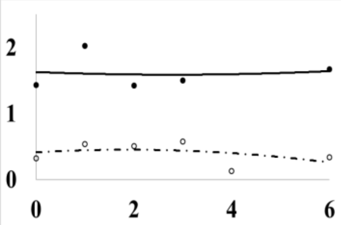	$y=0.0052x^2-0.029x+1.627$; $R^2=0.01$ (W)<br>$y=-0.0114x^2+0.0425x+0.4112$; $R^2=0.18$ (S)
**Lettuce+Cabbage**	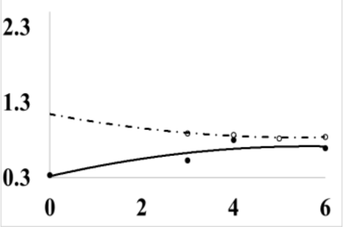	$y=-0.0124x^2+0.1405x+0.3164$; $R^2=0.80$ (W)<br>$y=0.0107x^2-0.1155x+1.1422$; $R^2=0.86$ (S)	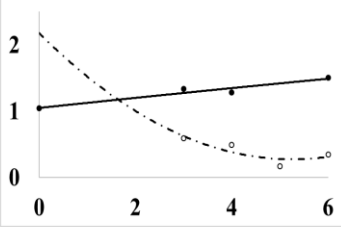	$y=-0.0006x^2+0.0772x+1.0464$; $R^2=0.92$ (W)<br>$y=0.069x^2-0.725x+2.176$; $R^2=0.74$ (S)
**Lettuce+Endive/ radicchio**	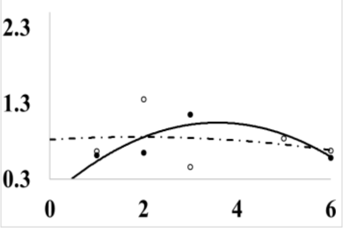	$y=-0.0768x^2+0.5493x+0.0607$; $R^2=0.70$ (W)<br>$y=-0.0103x^2+0.0389x+0.8188$; $R^2=0.05$ (S)	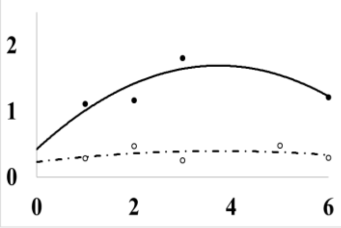	$y=-0.0908x^2+0.6788x+0.4219$; $R^2=0.68$ (W)<br>$y=-0.0119x^2+0.0892x+0.2263$; $R^2=0.09$ (S)
**Lettuce+Rocket**	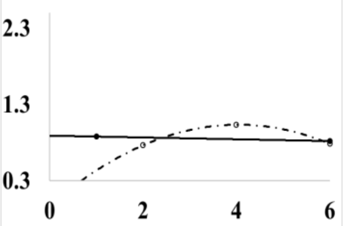	$y=-0.0118x+0.8886$; $R^2=1$ (W)<br>$y=-0.065x^2+0.5247x-0.0264$; $R^2=1$ (S)	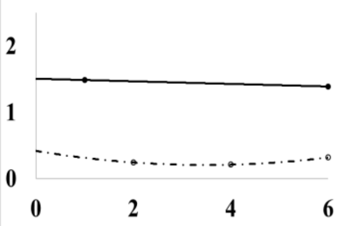	$y=-0.0195x+1.5045$; $R^2=1$ (W)<br>$y=0.0176x^2-0.1226x+0.4163$; $R^2=1$ (S)
**Lettuce+Chives**	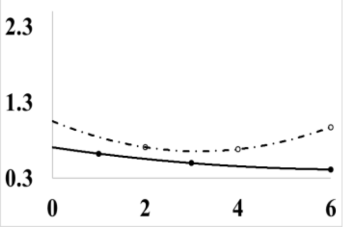	$y=0.0069x^2-0.0902x+0.7068$; $R^2=1$ (W)<br>$y=0.0398x^2-0.2518x+1.0533$; $R^2=1$ (S)	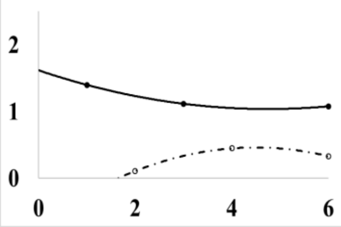	$y=0.0258x^2-0.2446x+1.6177$; $R^2=1$ (W)<br>$y=-0.0571x^2+0.512x-0.6877$; $R^2=1$ (S)
**Rocket**	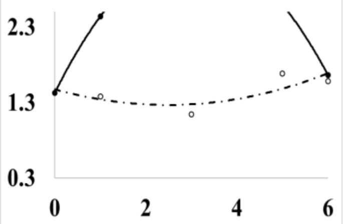	$y=-0.1945x^2+1.2065x+1.4263$; $R^2=1$ (W)<br>$y=0.034x^2-0.1682x+1.4739$; $R^2=0.59$ (S)	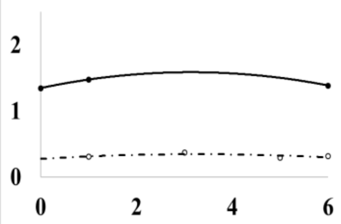	$y=-0.0246x^2+0.1539x+1.3441$; $R^2=1$ (W)<br>$y=-0.0065x^2+0.0426x+0.2732$; $R^2=0.37$ (S)
**Other**	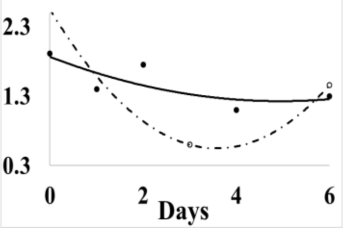	$y=0.0261x^2-0.2582x+1.8655$; $R^2=0.61$ (W)<br>$y=0.1558x^2-1.1159x+2.5471$; $R^2=1$ (S)	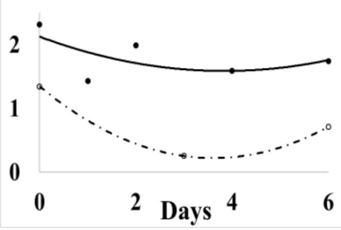	$y=0.0366x^2-0.2805x+2.1235$; $R^2=0.35$ (W)<br>$y=0.0863x^2-0.6234x+1.3442$; $R^2=1$ (S)

Figure S4. Effects of shelf life (days) on total phenolic content, antioxidants, % CO_2 and damage index (H_2O_2 and lipid peroxidation) per type of salad during winter (●, W) and summer (○, S).Other= Lettuce +2 or more ingredients.

	FRAP (mg trolox/g Fw)		ABTS (mg trolox/g Fw)	
**Lettuce**	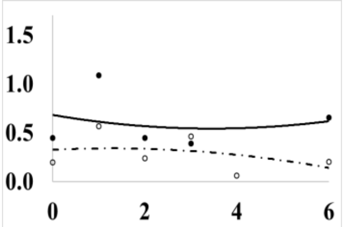	$y=0.0117x^2-0.081x+0.6813$; $R^2=0.03$ (W) $y=-0.0089x^2+0.0229x+0.3248$; $R^2=0.16$ (S)	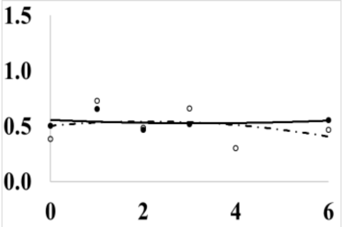	$y=0.003x^2-0.0191x+0.556$; $R^2=0.03$ (W) $y=-0.009x^2+0.038x+0.5028$; $R^2=0.10$ (S)
**Lettuce+Cabbage**	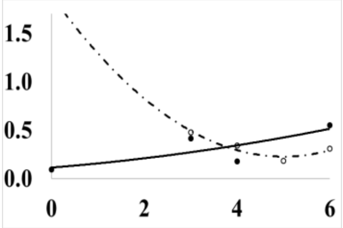	$y=0.0047x^2+0.0384x+0.1125$; $R^2=0.63$ (W) $y=0.0665x^2-0.6659x+1.8917$; $R^2=0.90$ (S)	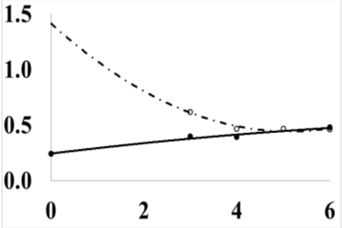	$y=-0.0021x^2+0.0512x+0.2427$; $R^2=0.97$ (W) $y=0.0369x^2-0.3787x+1.4153$; $R^2=0.93$ (S)
**Lettuce+Endive/ radicchio**	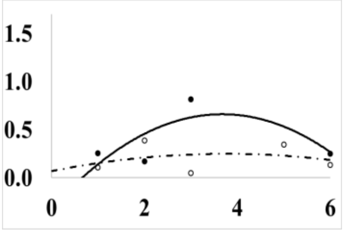	$y=-0.0726x^2+0.5335x-0.3188$; $R^2=0.51$ (W) $y=-0.0127x^2+0.0957x+0.0703$; $R^2=0.06$ (S)	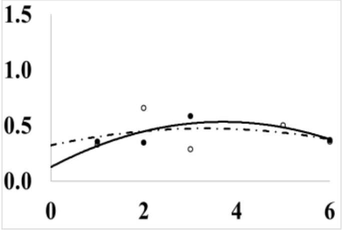	$y=-0.0297x^2+0.2198x+0.1248$; $R^2=0.59$ (W) $y=-0.0137x^2+0.0911x+0.3209$; $R^2=0.06$ (S)
**Lettuce+Rocket**	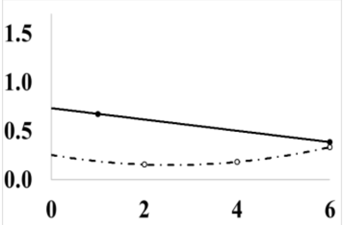	$y=-0.058x+0.7313$; $R^2=1$ (W) $y=0.0157x^2-0.0806x+0.2513$; $R^2=1$ (S)	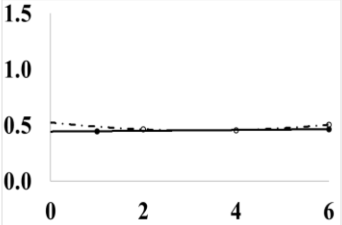	$y=0.0035x+0.4435$; $R^2=1$ (W) $y=0.0068x^2-0.0444x+0.5253$; $R^2=1$ (S)
**Lettuce+Chives**	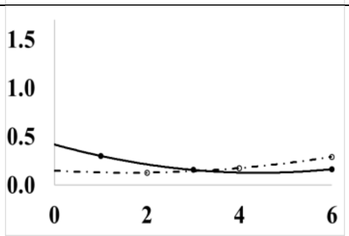	$y=0.0151x^2-0.1328x+0.4173$; $R^2=1$ (W) $y=0.0086x^2-0.028x+0.1488$; $R^2=1$ (S)	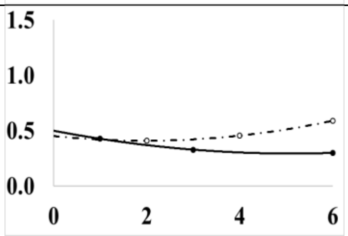	$y=0.0079x^2-0.0808x+0.4998$; $R^2=1$ (W) $y=0.0111x^2-0.0435x+0.4516$; $R^2=1$ (S)
**Rocket**	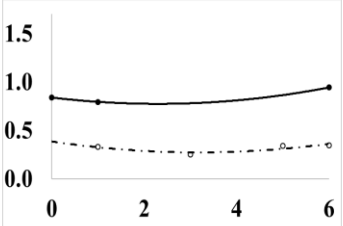	$y=0.0124x^2-0.057x+0.8376$; $R^2=1$ (W) $y=0.0112x^2-0.0713x+0.3847$; $R^2=0.73$ (S)	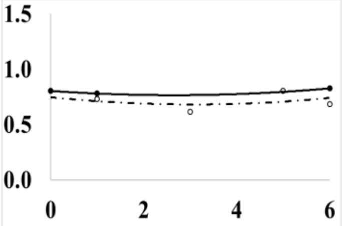	$y=0.0055x^2-0.0292x+0.8033$; $R^2=1$ (W) $y=0.0068x^2-0.0421x+0.7462$; $R^2=0.09$ (S)
**Other**	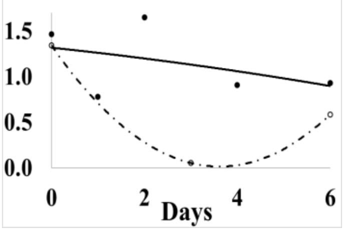	$y=-0.0027x^2-0.0536x+1.3158$; $R^2=0.19$ (W) $y=0.1012x^2-0.7333x+1.3412$; $R^2=1$ (S)	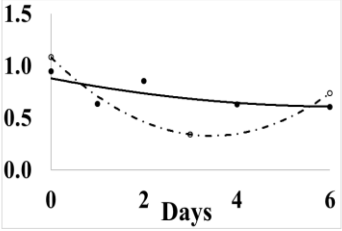	$y=0.0068x^2-0.0861x+0.8792$; $R^2=0.52$ (W) $y=0.0632x^2-0.4371x+1.0823$; $R^2=1$ (S)

Figure S4. (Continued)

	$\%CO_2$		H_2O_2 (µmol/g Fw)	
**Lettuce**	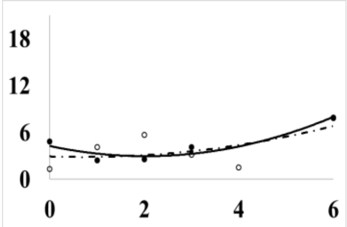	$y=0.3193x^2-1.293x+4.274$; $R^2=0.90$ (W)<br>$y=0.1421x^2-0.203x+2.9354$; $R^2=0.36$ (S)	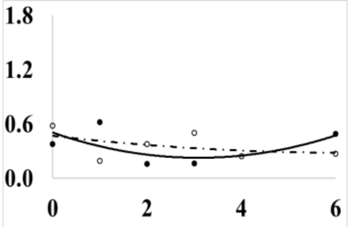	$y=0.0292x^2-0.1805x+0.5055$; $R^2=0.38$ (W)<br>$y=0.0048x^2-0.0596x+0.4689$; $R^2=0.20$ (S)
**Lettuce+Cabbage**	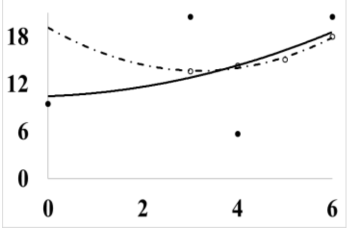	$y=0.1893x^2+0.2194x+10.432$; $R^2=0.20$ (W)<br>$y=0.5347x^2-3.4197x+19.13$; $R^2=0.98$ (S)	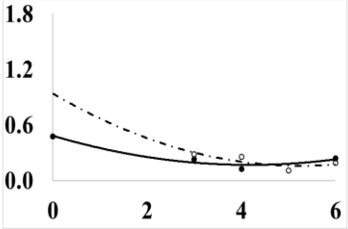	$y=0.0181x^2-0.1509x+0.4841$; $R^2=0.95$ (W)<br>$y=0.0283x^2-0.2974x+0.94$; $R^2=0.67$ (S)
**Lettuce+Endive/ radicchio**	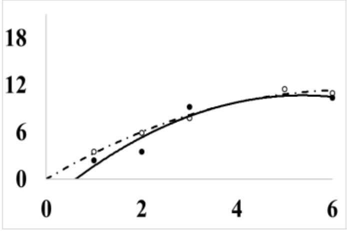	$y=-0.472x^2+5.0693x-2.9583$; $R^2=0.89$ (W)<br>$y=-0.2781x^2+3.5527x-0.0031$; $R^2=0.98$ (S)	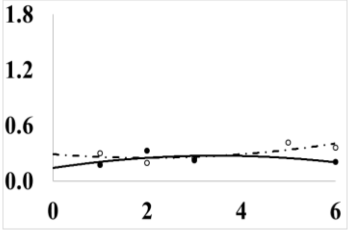	$y=-0.0111x^2+0.077x+0.1404$; $R^2=0.27$ (W)<br>$y=0.0097x^2-0.0388x+0.2897$; $R^2=0.57$ (S)
**Lettuce+Rocket**	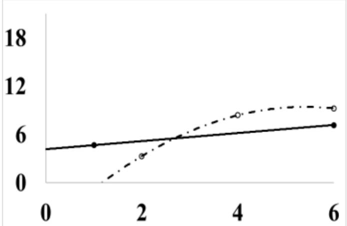	$y=0.502x+4.158$; $R^2=1$ (W)<br>$y=-0.5253x^2+5.6869x-5.9625$; $R^2=1$ (S)	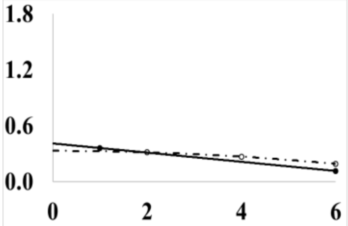	$y=-0.0492x+0.409$; $R^2=1$ (W)<br>$y=-0.0039x^2+0.0001x+0.3294$; $R^2=1$ (S)
**Lettuce+Chives**	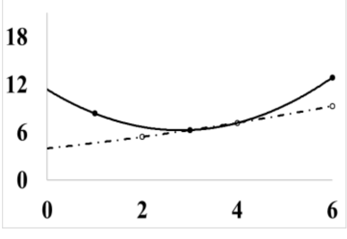	$y=0.6507x^2-3.6527x+11.372$; $R^2=1$ (W)<br>$y=0.0406x^2+0.6438x+3.965$; $R^2=1$ (S)	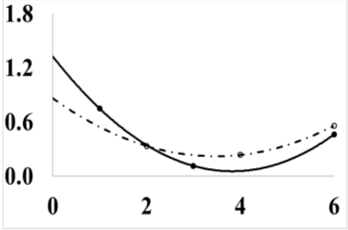	$y=0.0866x^2-0.6639x+1.3252$; $R^2=1$ (W)<br>$y=0.0532x^2-0.3693x+0.861$; $R^2=1$ (W)
**Rocket**	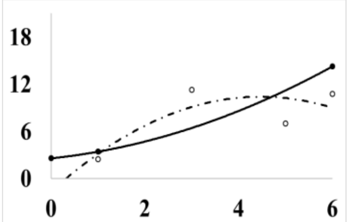	$y=0.2208x^2+0.6242x+2.555$; $R^2=1$ (W)<br>$y=-0.6042x^2+5.4043x-1.6783$; $R^2=0.62$ (S)	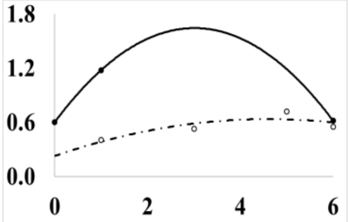	$y=-0.1148x^2+0.6913x+0.6017$; $R^2=1$ (W)<br>$y=-0.0193x^2+0.1774x+0.2264$; $R^2=0.73$ (S)
**Other**	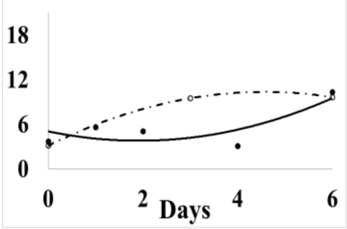	$y=0.343x^2-1.313x+5.0301$; $R^2=0.65$ (W)<br>$y=-0.3465x^2+3.1683x+3.1033$; $R^2=1$ (S)	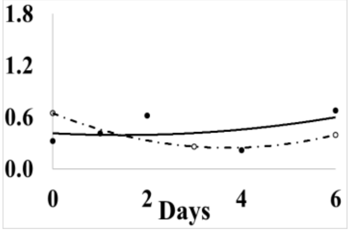	$y=0.0101x^2-0.0294x+0.4121$; $R^2=0.20$ (W)<br>$y=0.0291x^2-0.2164x+0.6452$; $R^2=1$ (S)

Figure S4. (Continued)

	MDA (nmol/g Fw)	
**Lettuce**	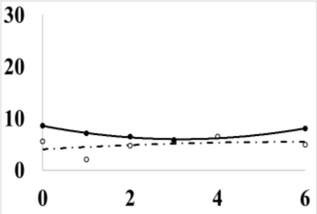	$y=0.2601x^2-1.6429x+8.567$;<br>$R^2=0.99$ (W)<br>$y=-0.0423x^2+0.4921x+4.0235$;<br>$R^2=0.13$ (S)
**Lettuce+Cabbage**	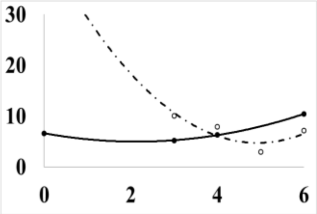	$y=0.3637x^2-1.5484x+6.615$; $R^2 = 1$ (W)<br>$y=1.5875x^2-15.648x+43.292$;<br>$R^2=0.74$ (S)
**Lettuce+Endive/ radicchio**	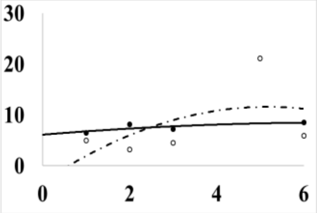	$y=-0.052x^2+0.7069x+6.1172$;<br>$R^2=0.59$ (W)<br>$y=-0.5544x^2+5.7334x-3.2237$;<br>$R^2=0.29$ (S)
**Lettuce+Rocket**	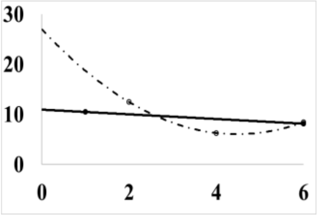	$y=-0.4685x+10.945$; $R^2=1$ (W)<br>$y=1.0445x^2-9.3578x+27.012$;<br>$R^2=1$ (S)
**Lettuce+Chives**	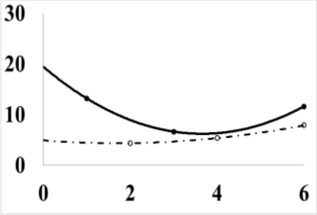	$y=0.9881x^2-7.2301x+19.413$;<br>$R^2=1$ (W)<br>$y=0.1919x^2-0.6568x+4.8891$;<br>$R^2=1$ (S)
**Rocket**	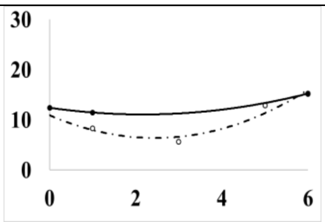	$y=0.2841x^2-1.2304x+12.423$;<br>$R^2=1$ (W)<br>$y=0.7599x^2-3.7156x+10.945$;<br>$R^2=0.93$ (S)
**Other**	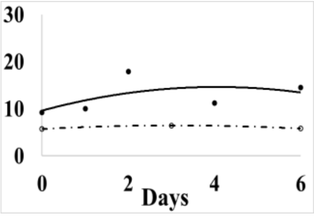	$y=-0.3059x^2+2.4755x+9.6229$;<br>$R^2=0.29$ (W)<br>$y=-0.0791x^2+0.4891x+5.7124$;<br>$R^2=1$ (S)

Figure S4. (Continued)
